# A DFT‐Based Protocol for Modeling the Structure and Reactivity of Gold(III) Complexes

**DOI:** 10.1002/jcc.70179

**Published:** 2025-07-09

**Authors:** Luana P. P. Cunha, Larissa P. N. M. Pinto, Willian T. G. Novato, Hélio F. Dos Santos, Diego F. S. Paschoal

**Affiliations:** ^1^ NQTCM: Núcleo de Química Teórica e Computacional de Macaé, Polo Ajuda, Instituto Multidisciplinar de Química, Centro Multidisciplinar UFRJ‐Macaé Universidade Federal do Rio de Janeiro Macaé Rio de Janeiro Brazil; ^2^ NEQC: Núcleo de Estudos em Química Computacional, Departamento de Química ‐ ICE Universidade Federal de Juiz de Fora, Campus Universitário Juiz de Fora Minas Gerais Brazil

**Keywords:** basis set, computational protocols, DFT, gold complexes, relativistic effects

## Abstract

In this study, distinct computational protocols were employed to investigate the structure and kinetic properties of the aquation reaction of the [Au(dien‐H)Cl]^+^ Au(III) complex. A total of 154 protocols with nonrelativistic Hamiltonians were initially assessed, comprising 31 basis sets for Au, 52 basis sets for ligand atoms, and 71 levels of theory (including HF, MP2, and 69 DFT‐functionals). Additionally, seven protocols with relativistic Hamiltonians, using all‐electron basis sets for Au, were evaluated. The results indicate that the structure is relatively insensitive to the computational protocol. In contrast, the activation Gibbs free energy (${\Delta G}_{\mathrm{aq}}^{‡}$) are highly sensitive to both the level of theory and basis sets choice. Notably, the basis set used for ligand atoms plays a key role in accurately predicting kinetic parameters. Among the tested 397 combinations, the B3LYP/def2‐SVP/6‐31G(d,p) protocol yielded the overall best agreement with experimental data for the reference complex. However, for bulkier [Au(R‐dien‐H)Cl]^+^ derivatives, diffuse functions on ligand atoms are essential, making 6‐31+G(d) the recommended basis set. When all five Au(III) complexes are considered, the optimal performance is achieved using B3LYP with the Stuttgart‐RSC ECP for Au and 6‐31+G(d) for ligand atoms. This combination offers a good balance between accuracy and computational cost, making it a practical choice even for larger Au(III) complexes.

## Graphical abstract

Computational protocols for predicting the ${\Delta G}_{\mathrm{aq}}^{‡}$ for the aquation reaction of gold complexes of the type [Au(R‐dien‐H)Cl]^+^ were proposed with the protocol B3LYP/Stuttgart‐RSC/6‐31+G(d) presenting a mean relative deviation (five Au(III) complexes) of only 4.0% in relation to the experimental values.

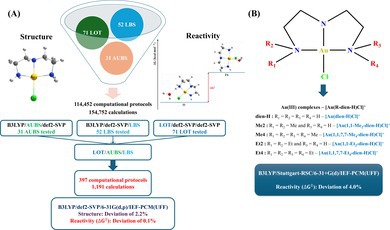

## 1 Introduction

Gold compounds have been used in human therapies since ancient Egypt to the present‐days. Although they have already been used for tuberculosis treatment, currently Au compounds are limited to the treatment of rheumatoid arthritis [1, 2, 3]. Nonetheless, there is a vast literature dedicated to their catalytic potential [4, 5] and, mainly, antitumoral properties [5, 6, 7, 8, 9]. Thus, Au complexes appear as an alternative to the main anticancer metallodrug cisplatin, with promising effectiveness against cisplatin‐resistant cancer cells [1, 10, 11, 12, 13, 14, 15].

Au(III) ion has a d^8^ electronic configuration, and its coordination complexes present square‐planar geometry, like isoelectronic and isostructural Pt(II) complexes, which are well recognized as important anticancer drugs. However, under physiological conditions, Au(III) is rapidly hydrolyzed and reduced to Au(I). Thus, stabilization of Au(III) complexes with suitable ligands, such as C^N^C and N^N^N ligands, is desirable [2, 10, 16]. Most of the available studies suggest that proteins and/or enzymes containing thiol groups should play a significant role in the mechanism of action of potential anticancer Au complexes. The Au(III) complexes may reduce to Au(I) and undergo ligand‐exchange reactions, activating the complexes to act with high affinity to thiol and selenol groups, such as those present in the thioredoxin reductase (TrxR) enzyme [16, 17, 18, 19].

**FIGURE 1:**
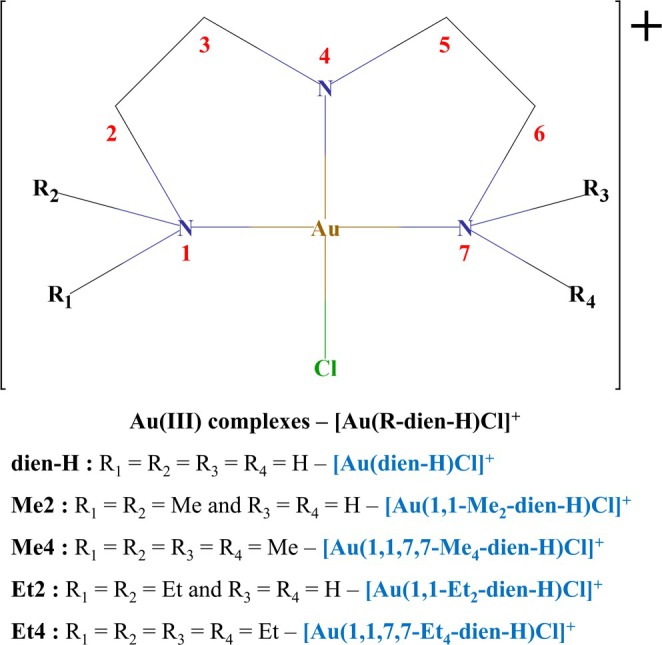
Structure of the five [Au(R‐dien‐H)Cl]^+^ complexes analyzed in this study. The atom numbering scheme used for structural analysis is also shown.

Given the interest in the antitumoral properties of Au(III) complexes, computational studies are important to evaluate the reactivity of the compounds against biological targets [20, 21, 22, 23, 24]. Zhao and Zhou [20] described the reactions of [Au(DMDT)(Cl)(H_2_O)]^+^ and [Au(DMDT)(H_2_O)_2_]^2+^ complexes with cysteine and DNA purine bases at B3LYP/LANL2DZ/6‐311++G(2d,2p)//B3LYP/LANL2DZ/6‐31(d,p) level. The authors showed that the Au(III) complex has more favorable kinetics with cysteine nitrogen compared to sulfur, and that guanine is a preferred biological target when compared to cysteine. The kinetics of the substitution reactions for Au(III) complexes with biologically relevant ligands were studied by Djekovic et al. [21] at B3LYP/LANL2DZp level. From the calculated results, the authors concluded that the reactions of the studied complexes have an interchange mechanism with an associative character (*I*_a_). In both studies, the authors carried out geometry optimization and vibrational frequencies calculations in the gas phase, including the solvent only for energy correction. Besides, the authors did not compare the results directly with the experimental data. In that same period, we studied the ligand exchange reaction of [Au(R‐dien)Cl]^2+^ and [Au(R‐dien‐H)Cl]^+^ complexes with H_2_O and other strong nucleophiles at B3LYP/Stuttgart‐RSC+1f(*α* = 1.14)/6‐31+G(d)/IEF‐PCM(UFF) level [22, 23]. The results were compared directly with experimental data, showing a satisfactory agreement for the reaction of the [Au(dien‐H)Cl]^+^ with H_2_O ($k^{\text{calc}.}$ = 0.36 s^−1^ and $k^{\text{expt}.}$ = 0.6 s^−1^). Although good results have been obtained in these previous studies, they lack a systematic benchmarking focused on the computational protocol, which could generalize and broaden its application for Au(III)‐complexes. In the present paper, we report a systematic benchmarking including basis sets, level of theory, relativistic effects, and empirical dispersion corrections to calculate the geometry and kinetic properties for aquation reaction of the [Au(dien‐H)Cl]^+^ [25, 26] complex and four alkylated derivatives, [Au(R‐dien‐H)Cl]^+^ [27] (Figure 1), for which experimental data are available.

## 2 Theoretical Methodology

**FIGURE 2:**
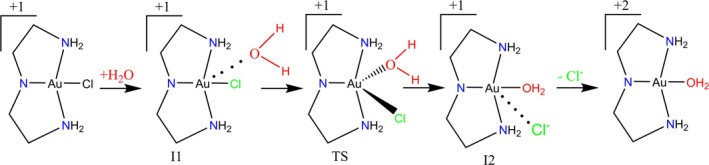
Aquation reaction of [Au(dien‐H)Cl]^+^ complex.

The aquation reaction of the [Au(dien‐H)Cl]^+^ complex is shown in Figure 2. The geometries of all reactive species were optimized and characterized as a minimum or transition state (TS) on the potential energy surface (PES) through harmonic frequency calculations. The TS structure was initially guessed as a distorted trigonal bipyramidal and characterized by the existence of only one imaginary frequency (a saddle point on the PES), with the vibrational normal mode representing the break and the formation of bonds at the equatorial region. From the TS structure, the intrinsic reaction coordinate (IRC) was followed through 20 points with 0.1 a.m.u^1/2^ Bohr step size connecting the TS to the reagent and product minima. The structure of these minimum points, called intermediates 1 (I1‐reagent) and 2 (I2‐product), were further optimized to ensure the full geometry convergence. The integral equation formalism for the polarizable continuum model (IEF‐PCM) [28], using the dielectric constant ε = 78.3553 (water) and the UFF set of atomic radii, was used to account for the solvent effect in the structure and energies. With all these data, the activation Gibbs free energy (${\Delta G}_{\mathrm{aq}}^{‡}$, Equation (1)) and the corresponding rate constant (k, Equation (2)) were calculated for the direct reaction (I1 → TS). These first calculations were performed at Density Functional Theory (DFT) level with B3LYP functional [29, 30, 31] and def2‐SVP basis sets [32, 33] (B3LYP/def2‐SVP/def2‐SVP protocol). For Au atom, the def2‐SVP basis set explicitly treats the 19 outermost (valence) electrons and considers the effective core potential (ECP) def2‐ECP, which is a modified version of the Stuttgart/Dresden (SDD) ECP and which considers the scalar relativistic effects, for 60 core electrons (AOs 1s to 4f) [32, 33].

The $∆G_{\mathrm{aq}}^{‡}$ was calculated according to Equation (1) [34]:

(1) $$∆G_{\mathrm{aq}}^{‡}=G_{\mathrm{aq}}\left(\mathrm{TS}\right)-G_{\mathrm{aq}}\left(I1\right)$$

Subsequently, the k was calculated using the Eyring‐Polanyi Equation (2) at standard conditions of *T* = 298.15 K, c^0^ = 1 mol L^−1^ and *p* = 1 atm.

(2) $$k=\frac{k_{B}T}{{\mathrm{hc}}^{0}}\exp\left(\frac{-∆G_{\mathrm{aq}}^{‡}}{\mathrm{RT}}\right)$$

with *k*_B_, *h*, and *R* being the Boltzmann, Planck, and ideal gas constants, respectively (*c* ^0^ = 1 mol L^−1^).

The benchmarking procedure included geometry optimization and harmonic frequency calculations for the TS and I1 species at all protocols evaluated. To assess the performance of several computational protocols, we initially selected 31 Au basis sets (AUBS), including effective core potentials (ECP), which consider the scalar relativistic effects in the potential employed in the description of the 60 core electrons, and all‐electron nonrelativistic (NR) basis sets (ABS) (Table 1), 52 NR ligands basis sets (LBS) (Table 2), and the levels of theory (LOT) HF, MP2, and DFT (using 48 distinct density functionals) (Table 3). In addition, the dispersion corrections were also considered through the empirical approximations D3 with the original damping function (11 density functionals) and D3BJ with Becke‐Johnson damping (10 density functionals) (see also Table 3). Considering all possible combinations, 114,452 computational protocols can be proposed, which implies 343,356 calculations (complex structure, TS, and I1). The huge number of protocols to be tested is prohibitive for practical reasons and would lead to many similar results due to the subtle changes between them. Thus, we proposed a screening of methods based on three sets of calculations: B3LYP/AUBS/def2‐SVP, B3LYP/def2‐SVP/LBS, and LOT/def2‐SVP/def2‐SVP, resulting in 154 distinct protocols and 462 calculations needed, including all NR Hamiltonian methods. All these calculations were performed with the Gaussian 09 Rev. D.01 program [51].

**TABLE 1:** Au basis sets (AUBS) used for benchmarking, including effective core potentials (ECP), nonrelativistic, and relativistic all‐electron basis sets (ABS).

ECP (60 electrons)	Basis set	Contraction scheme^r^	GTO	CGTO
	Basis set (19 electrons)	(Uncontracted) → [Contracted]		
MCDHF^a^	cc‐pVDZ‐PP^a^	(22s19p11d1f) → [4s4p3d1f]	141	38
	cc‐pVTZ‐PP^a^	(37s33p22d2f1g) → [5s5p4d2f1g]	269	63
	cc‐pVQZ‐PP^a^	(66s51p37d3f2g1h) → [6s6p5d3f2g1h]	454	99
	cc‐pV5Z‐PP^a^	(91s73p51d4f3g2h1i) → [7s7p6d4f3g12h1i]	655	258
	cc‐pwCVDZ‐PP^a^	(23s20p12d2f) → [5s5p4d2f]	157	54
	cc‐pwCVTZ‐PP^a^	(38s34p23d3f2g) → [6s6p5d3f2g]	294	88
	cc‐pwCVQZ‐PP^a^	(67s52p38d4f3g2h) → [7s7p6d4f3g2h]	490	135
	cc‐pwCV5Z‐PP^a^	(93s75p53d5f4g3h2i) → [9s9p8d5f4g3h2i]	713	206
	aug‐cc‐pVDZ‐PP^a^	(23s20p12d2f) → [5s5p4d2f]	157	54
	aug‐cc‐pVTZ‐PP^a^	(38s34p23d3f2g) → [6s6p5d3f2g]	294	88
	aug‐cc‐pVQZ‐PP^a^	(67s52p38d4f3g2h) → [7s7p6d4f3g2h]	490	135
	aug‐cc‐pV5Z‐PP^a^	(92s74p52d5f4g3h2i) → [8s8p7d5f4g3h2i]	704	197
AREP^b^	CRENBL^b^	(5s5p4d) → [5s5p4d]	40	40
def2‐ECP^c^, ^d^	def2‐SV^d^	(7s6p5d) → [6s3p2d]	50	25
	def2‐SVP^d^	(7s6p5d1f) → [6s3p2d1f]	57	32
	def2‐SVPD^e^	(7s7p5d1f) → [6s4p2d1f]	60	35
	def2‐TZVP^d^	(8s7p6d1f) → [6s4p3d1f]	66	40
	def2‐TZVPP^d^	(8s7p6d2f1g) → [6s4p3d2f1g]	82	56
	def2‐TZVPD^e^	(8s8p6d1f) → [6s5p3d1f]	69	43
	def2‐TZVPPD^e^	(8s8p6d2f1g) → [6s5p3d2f1g]	85	59
	def2‐QZVP^d^	(10s8p6d3f1g) → [7s5p4d3f1g]	94	72
	def2‐QZVPP^d^	(10s8p6d4f2g) → [7s5p4d4f2g]	110	88
Hay‐Wadt^f^	LANL2DZ^f^	(8s6p3d) → [3s3p2d]	41	22
	modified‐LANL2DZ^g^	(8s8p3d) → [3s3p2d]	47	22
	LANL2TZ^h^	(5s5p3d) → [5s5p3d]	35	35
	LANL2TZ(f)^i^	(5s5p3d1f) → [5s5p3d1f]	42	42
RCEP^j^	SBKJC‐VDZ^j^	(7s7p5d) → [4s4p3d]	53	31
SDD^k^	Stuttgart‐RSC^l^	(8s6p5d) → [7s3p4d]	51	36
	Stuttgart‐RSC(f)^l^, ^m^	(8s6p5d1f) → [7s3p4d1f]	58	43
*Nonrelativistic ABS*				
—	jorge‐DZP^n^	(20s16p9d5f) → [8s7p4d2f]	148	63
—	jorge‐ADZP^n^	(21s17p10d6f) → [9s8p5d3f]	164	79
—	jorge‐TZP^o^	(21s15p10d6f1g) → [9s6p5d3f1g]	167	82
—	jorge‐ATZP^o^	(21s16p11d7f2g) → [9s7p6d4f2g]	191	106
*Relativistic ABS*				
—	jorge‐DZP‐DKH^n^	(20s16p9d5f) → [8s7p4d2f]	148	63
—	jorge‐TZP‐DKH^o^	(21s15p10d6f1g) → [9s6p5d3f1g]	167	82
—	Sapporo‐DKH3‐DZP‐2012^p^	(24s18p14d10f) → [8s6p5d2f]	218	65
—	Sapporo‐DKH3‐DZP‐2012‐diffuse^p^	(25s19p15d11f) → [9s7p6d3f]	234	81
—	Sapporo‐DKH3‐TZP‐2012^p^	(25s20p15d10f2g) → [10s8p6d3f1g]	248	94
—	Sapporo‐DKH3‐TZP‐2012‐diffuse^p^	(26s21p16d11f3g) → [11s9p7d4f2g]	273	119
—	SARC‐DKH2^q^	(22s15p11d6f) → [17s11p8d2f]	164	104

Abbreviations: AREP, Ab initio averaged relativistic effective core potential; def2‐ECP, Modified version of the Stuttgart/Dresden ECP; MCDHF, Fully relativistic four‐component multiconfiguration Dirac‐Hartree‐Fock (MCDHF) ECP; RCEP, Relativistic compact effective potential; SDD, Relativistic Stuttgart/Dresden ECP.

^a^ From reference [35].

^b^ From reference [36].

^c^ From references [32, 33].

^d^ From reference [33].

^e^ From reference [37].

^f^ From reference [38].

^g^ From reference [39].

^h^ From reference [40].

^i^ From references [40, 41].

^j^ From reference [42].

^k^ From reference [32].

^l^ From reference [43].

^m^ From reference [22, 43].

^n^ From reference [44].

^o^ From reference [45].

^p^ From reference [46].

^q^ From reference [47]. All ECP used are relativistic and describe the 60 core electrons of Au (1s to 4f). The ECP + basis sets used in the present work were obtained in the Basis Set Exchange Portal (https://www.basissetexchange.org/) [48, 49, 50] under the name of the basis set. The ABS basis sets were also obtained in the Basis Set Exchange Portal (https://www.basissetexchange.org/) [48, 49, 50].

^r^ The basis sets are listed according to the format adopted by the GAUSSIAN program [51], used in this study.

**TABLE 2:** Gaussian basis sets used for ligands atoms (H, C, N, O, and Cl).

Double‐zeta	Triple‐zeta	Quadruple‐Zeta	Type
*Nonrelativistic*			
6‐31G	6‐311G		Pople^a^
6‐31 + G	6‐311 + G		
6‐31++G	6‐311++G		
6‐31G(d)	6‐311G(d)		
6‐31 + G(d)	6‐311 + G(d)		
6‐31++G(d)	6‐311++G(d)		
6‐31G(d,p)	6‐311G(d,p)		
6‐31 + G(d,p)	6‐311 + G(d,p)		
6‐31++G(d,p)	6‐311++G(d,p)		
6‐31 + G(2d)	6‐311 + G(2d)		
6‐31 + G(2d,p)	6‐311 + G(2d,p)		
6‐31 + G(2df)	6‐311 + G(2df)		
6‐31 + G(2df,p)	6‐311 + G(2df,p)		
6‐31 + G(2df,2p)	6‐311 + G(2df,2p)		
6‐31++G(2df,2pd)	6‐311++G(2df,2pd)		
cc‐pVDZ	cc‐pVTZ	cc‐pVQZ	Dunning^b^
aug‐cc‐pVDZ	aug‐cc‐pVTZ	aug‐cc‐pVQZ	
jorge‐DZP	jorge‐TZP	jorge‐QZP	Jorge^c^
jorge‐ADZP	jorge‐ATZP	jorge‐AQZP	
def2‐SVP	def2‐TZVP	def2‐QZVP	Ahlrichs^d^
def2‐SVPD	def2‐TZVPD	def2‐QZVPD	
Sapporo‐DZP‐2012	Sapporo‐TZP‐2012		Sapporo^e^
Sapporo‐DZP‐2012‐diffuse	Sapporo‐TZP‐2012‐diffuse		
*Relativistic*			
jorge‐DZP‐DKH			Jorge^f^

^a^ From the references [52, 53, 54, 55, 56, 57, 58, 59, 60, 61].

^b^ From the references [62, 63, 64].

^c^ From the references [65, 66, 67].

^d^ From the references [33, 37, 68].

^e^ From the references [69, 70, 71].

^f^ From the reference [72]. All basis sets were obtained in Basis Set Exchange Portal (https://www.basissetexchange.org/) [48, 49, 50].

**TABLE 3:** Ab initio methods and DFT functionals used for benchmarking.

Ab initio	LDA	GGA	Meta‐GGA	Hybrid
HF [73, 74, 75]	SVWN [76, 77, 78]	mPWLYP [29, 79, 80]	VSXC [81]	B972 [82]
MP2 [83, 84, 85, 86, 87]		mPWPBE [79, 88, 89]	BB95 [90, 91]	mPW1LYP [29, 79, 80]
		mPWPW91 [79, 92, 93, 94]	TPSS [95]	mPW3PBE [79, 94]
		OLYP [29, 80, 96, 97]	M06L [98]	mPW1PW91 [79, 99]
		BLYP [29, 80, 90]	M11L [100]	O3LYP [101]
		XLYP [29, 76, 77, 80]	MN12L [102]	X3LYP [103]
		BPW91 [90, 92, 93, 94]	MN15L [104]	B3LYP [29, 30, 31]
		BP86 [90, 105]		B3PW91 [31, 92]
		PBE [88, 89]		B3P86 [31, 105]
		τ‐HCTH [106]		PBE0 [107, 108]
		SOGGA11 [109]		BHandH [110]
				BHandHLYP [110]
				SOGGA11X [111]

Hybrid meta‐GGA	LRC	D3 [112]	D3BJ [113]
BMK [114]	ωB97 [115]	BLYP‐D3	BLYP‐D3BJ
τHCTHhyb [106]	ωB97XD [116]	BP86‐D3	BP86‐D3BJ
B1B95 [91]	LC‐BLYP [29, 80, 90, 117]	PBE‐D3	PBE‐D3BJ
TPSSh [95, 118, 119]	LC‐ωPBE [120, 121, 122]	B97‐D3	B97‐D3BJ
PW6B95 [123]	CAM‐B3LYP [124]	TPSS‐D3	TPSS‐D3BJ
M06‐HF [125, 126]	M11 [109]	M06‐D3	B3LYP‐D3BJ
M06 [127]	MN12SX [128]	B3LYP‐D3	B3PW91‐D3BJ
M06‐2X [127]		B3PW91‐D3	PBE0‐D3BJ
MN15 [129]		PBE0‐D3	CAM‐B3LYP‐D3BJ
		CAM‐B3LYP‐D3	LC‐ωPBE‐D3BJ
		LC‐ωPBE‐D3	

Abbreviation: LRC, Long‐range corrected.

The role of including relativistic effects in the Hamiltonian was assessed through two B3LYP/AUBS/jorge‐DZP/C‐PCM and seven B3LYP‐DKH2/AUBS/jorge‐DZP‐DKH/C‐PCM computational schemes, in which the scalar relativistic 2nd‐order Douglas‐Kroll‐Hess (DKH2) approach [130, 131, 132, 133, 134] and the conductor‐like Continuum Polarization Model (C‐PCM) [135] were used as implemented in the ORCA 4.2.1 program [136]. Lastly, the best‐performing protocols were tested for four alkylated derivatives of the reference molecule, [Au(R‐dien‐H)Cl]^+^, where *R* = CH_3_ and CH_3_CH_2_ (Figure 1).

## 3 Results and Discussion

### 3.1 Structure Analysis for the [Au(dien‐H)Cl]^+^ Reference Molecule

Initially, the role of AUBS, LBS, and LOT to predict the structure of the [Au(dien‐H)Cl]^+^ complex (Figure 1) was assessed using the X‐ray data as reference [26]. The Au−N1, Au−N4, and Au−Cl bond lengths and N1−Au−N7, N1−Au−N4, N1−Au−Cl, and N4−Au−Cl bond angles were analyzed. To quantify the protocol's performance, the relative deviation (Equation 3) and mean relative deviation (Equation 4—MRD) were used [137].

(3) $$\delta_{i,j}=\frac{s_{i}^{\text{expt}}-s_{i,j}^{\text{calc}}}{s_{i}^{\text{expt}}}\times 100$$

(4) $$\mathrm{MRD}=\frac{1}{n_{k}}\sum_{k=1}^{n_{k}}\left|\delta_{i,j}\right|$$

where *i* refers to the structural parameter and *j* to the computational protocol used. For example, δ_Au‐N2,B3LYP/def2‐SVP/def2‐SVP_ is the error found for Au−N2 bond length at B3LYP(LOT)/def2‐SVP(AUBS)/def2‐SVP(LBS) level. In Equation (4), $n_{k}=7$ is the number of structural parameters evaluated.

The absolute values of structural parameters and the MRD are presented in Tables S1–S4 and summarized in Table 4. According to Table 4, the structure of the probe complex [Au(dien‐H)Cl]^+^ is well reproduced by most of the computational protocols tested, with an average MRD of only 2.0%. This corresponds to a maximum deviation of 0.09 Å for the Au‐Cl bond (3.7%) and 3.47° for the N1‐Au‐N4 bond angle (4.0%). Analysis within the individual protocol sets reveals that the quintuple‐zeta AUBS cc‐pwCV5Z‐PP yielded the lowest MRD (1.5%) when combined with B3LYP and the def2‐SVP LBS. For comparisons within the LBS set, keeping B3LYP and def2‐SVP for Au, the best agreement with experimental structure was achieved using the aug‐cc‐pVTZ basis set, resulting in an MRD of 1.9%. The Au−Cl bond was more sensitive to the LBS than Au−N bonds due to the chloride ligand, in which polarization d‐functions are essential to represent chemical bonds. For instance, the addition of the first set of polarization functions to the LBS changes the Au−Cl from 2.463 (6‐31G) to 2.418 Å(6‐31G(d)), with the latter in better agreement with the experiment (2.333 Å). The agreement with the experimental structure is further improved with the addition of the second set of polarization functions (d and f) and diffuse functions, as represented by 6‐31+G(2df) (2.404 Å). The basis sets analysis suggests a main role of AUBS over LBS and that achieving a balance between these two components of the protocol would be desirable in order to set up an affordable computational cost, that is, large valence basis set for Au is necessary to compensate for the relatively small basis set used for the ligands.

**TABLE 4:** Average deviation and MRD calculated for structural parameters of [Au(dien‐H)Cl]^+^ complex.

	AUBS^a^	LBS^b^	LOT^c^	LOT‐dispersion^d^
Au‐N1/Å	0.06	0.04	0.03	0.04
Au‐N4/Å	−0.04	−0.03	−0.05	−0.04
Au‐Cl/Å	0.06	0.09	0.05	0.05
N1‐Au‐N7/°	−2.39	−2.15	−2.01	−2.42
N1‐Au‐N4/°	−2.12	−3.47	−3.41	−3.46
N1‐Au‐Cl/°	2.16	1.42	1.38	1.49
N4‐Au‐Cl/°	−1.31	−1.25	−1.69	−1.79
MRD/%	2.0	2.1	2.0	2.0

^a^ B3LYP/AUBS/def2‐SVP/IEF‐PCM(UFF) (31 protocols).

^b^ B3LYP/def2‐SVP/LBS/IEF‐PCM(UFF) (52 protocols).

^c^ LOT/def2‐SVP/def2‐SVP/IEF‐PCM(UFF) (50 protocols).

^d^ LOT‐D3/def2‐SVP/def2‐SVP/IEF‐PCM(UFF) and LOT‐D3(BJ)/def2‐SVP/def2‐SVP/IEF‐PCM(UFF) (21 protocols).

The final set of protocols includes calculations performed at the LOT, LOT‐D3, and LOT‐D3(BJ) levels of theory. At the B3LYP/def2‐SVP/def2‐SVP level, the MRD was 2.2%, comparable to HF (2.1%) but higher than MP2 (1.5%). The best overall structural agreement was achieved using the MN15 functional, with an MRD of 1.4%. When dispersion corrections were included, the most accurate result was obtained at the LC‐ωPBE‐D3 level, with an MRD of 1.5%. These results suggest the important role of the chosen DFT functional on the predicted geometry of Au(III) complexes. Notably, very accurate structures (MRD = 1.4%) can be obtained even with relatively small basis sets (def2‐SVP) applied to both Au and ligands atoms. The 10 protocols that produced the lowest MRD values for the probe complex are listed in the left column of Table 5.

**TABLE 5:** Top 10 nonrelativistic computational protocols for predicting the structure and reactivity of the [Au(dien‐H)Cl]^+^ Au(III) complex.

Structure	MRD/%	Reactivity	RD/%
MN15/def2‐SVP/def2‐SVP	1.42	B3LYP/def2‐SVP/6‐31G(d,p)	0.10
M11/def2‐SVP/def2‐SVP	1.46	B3LYP/def2‐SVP/6‐31G(d)	0.18
MP2/def2‐SVP/def2‐SVP	1.47	TPSS‐D3BJ/def2‐SVP/def2‐SVP	0.64
B3LYP/cc‐pwCV5Z‐PP/def2‐SVP	1.48	BLYP‐D3BJ/def2‐SVP/def2‐SVP	0.97
SVWN/def2‐SVP/def2‐SVP	1.49	B3LYP/def2‐SVP/Sapporo‐DZP‐2012	1.04
B3LYP/aug‐cc‐pV5Z‐PP/def2‐SVP	1.51	B3LYP/def2‐SVP/cc‐pVDZ	1.09
B3LYP/aug‐cc‐pVQZ‐PP/def2‐SVP	1.51	B3LYP/def2‐SVP/jorge‐DZP	1.21
B3LYP/cc‐pV5Z‐PP/def2‐SVP	1.53	M11/def2‐SVP/def2‐SVP	2.63
M06‐HF/def2‐SVP/def2‐SVP	1.53	B3LYP/Stuttgart‐RSC/def2‐SVP	2.68
LC‐ωPBE‐D3/def2‐SVP/def2‐SVP	1.53	TPSS‐D3/def2‐SVP/def2‐SVP	2.92

### 3.2 Analysis of the Aquation Reaction for the [Au(dien‐H)Cl]^+^ Reference Molecule

Figure 2 represents the aquation reaction for [Au(dien‐H)Cl]^+^, in which the I1 → TS step is the focus of the present analysis. The ${∆G}_{\mathrm{aq}}^{‡}$ and the corresponding rate constant (k) were calculated and compared to the experimental values [25]: ${∆G}_{\mathrm{aq}}^{‡}=17.76\;\text{kcal}\;\mathrm{mo}l^{-1}$ and $k^{\text{expt}}=0.6\;M^{-1}\;s^{-1}$. The calculated absolute values for the properties can be found in Tables S5–S8.

**FIGURE 3:**
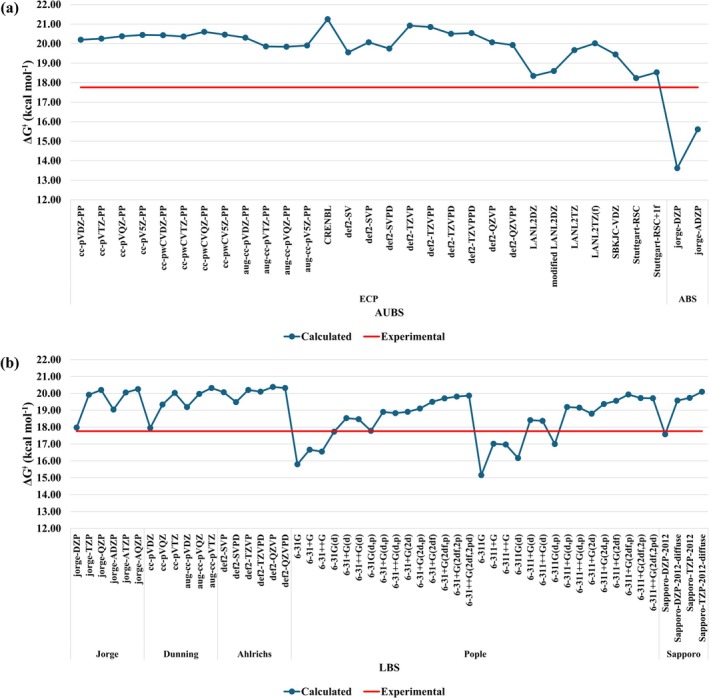
Calculated ${∆G}_{\mathrm{aq}}^{‡}$ (kcal mol^−1^) at (a) B3LYP/AUBS/def2‐SVP/IEF‐PCM(UFF) levels, (b) B3LYP/def2‐SVP/LBS/IEF‐PCM(UFF) levels. The red‐line represents the experimental value, ${∆G}_{\mathrm{aq}}^{‡}$ = 17.76 kcal mol^−*1*^ [25].

The role of the Au basis set was assessed first at B3LYP/AUBS/def2‐SVP/IEF‐PCM(UFF) level. The calculated ${∆G}_{\mathrm{aq}}^{‡}$ values are shown in Figure 3a. We note that the calculated values with ECP are overestimated by 12.5% (2.22 kcal mol^−1^) on average. The use of all‐electrons basis set (ABS) for Au decreases the barrier below the experimental value (Figure 3a). In general, the ECP leads to better results, with Stuttgart‐RSC and LANL2DZ providing the best agreement with experimental data, 18.24 and 18.34 kcal mol^−1^, respectively. These results were slightly worse than our previous calculation at B3LYP/Stuttgart‐RSC+1f/6‐31+G(d)/IEF‐PCM(UFF) [22], 18.04 kcal mol^−1^.

Figure 3b presents the calculated values of ${∆G}_{\mathrm{aq}}^{‡}$ at B3LYP/def2‐SVP/LBS/IEF‐PCM(UFF) level. Comparing Figure 3a,b we note that ${∆G}_{\mathrm{aq}}^{‡}$ is more sensitive to the LBS than AUBS. Figure 3b shows clearly that only few LBS provided satisfactory results, with relative error of ~1.0%, namely, jorge‐DZP (1.2%), cc‐pVDZ (1.1%), Sapporo‐DZP‐2012 (1.0%), 6‐31G(d) (0.2%), and 6‐31G(d,p) (0.1%). On average, the relative deviation was 8.8%, which corresponds to an average absolute deviation of 1.6 kcal mol^−1^, smaller than obtained for varying the AUBS (12.5% and 2.2 kcal mol^−1^ on average). It is interesting to note that the addition of one set of polarization functions is very important, with the error decreasing from 11.1% (6‐31G) to only 0.2% (6‐31G(d)). Therefore, at B3LYP, the best choice for AUBS and LBS is def2‐SVP and 6‐31G(d,p), respectively, which is an affordable computational protocol to modeling reactions of large Au(III) complexes. At this level, the calculated ${∆G}_{\mathrm{aq}}^{‡}$ was 17.78 kcal mol^−1^, in excellent accordance with the experimental value, 17.76 kcal mol^−1^.

**FIGURE 4:**
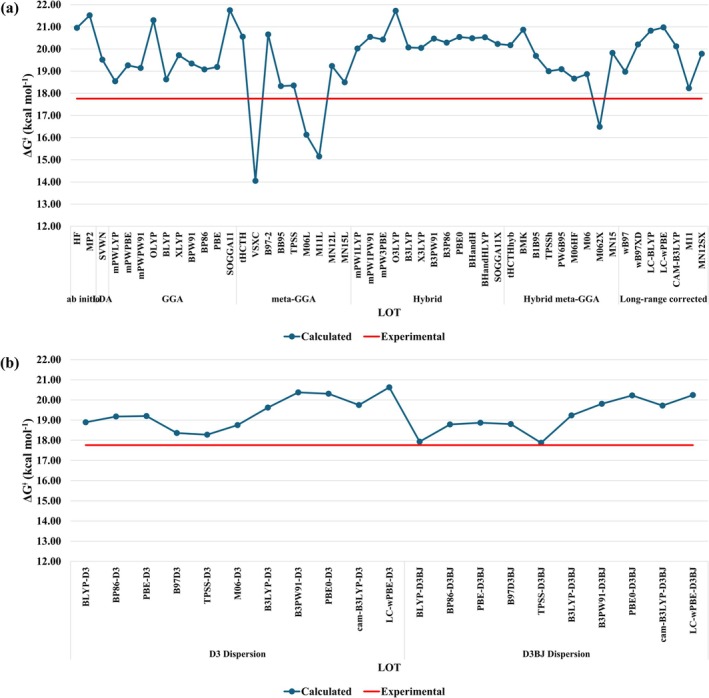
Calculated ${∆G}_{\mathrm{aq}}^{‡}$ (kcal mol^−1^) at (a) LOT/def2‐SVP/def2‐SVP/IEF‐PCM(UFF) levels, (b) LOT‐D3(BJ)/def2‐SVP/def2‐SVP/IEF‐PCM(UFF) levels. The red‐line represents the experimental value, ${∆G}_{\mathrm{aq}}^{‡}$ = 17.76 kcal mol^−*1*^ [25].

The role of the level of theory on the activation barrier was assessed using HF, MP2, and various DFT functionals, including those with empirical dispersion correction (D3 and D3(BJ)). The results are summarized in Figure 4. Overall, the data show a fairly scattered trend, with most protocols overestimating the activation barrier by an average of 2.1 kcal mol^−1^ without dispersion correction (Figure 4a) and 1.5 kcal mol^−1^ with D3(BJ) included (Figure 4b). Among the tested protocols, M11/def2‐SVP/def2‐SVP and TPSS‐D3BJ/def2‐SVP/def2‐SVP showed relatively low deviations (2.6% and 0.6%, respectively), though both were worse than B3LYP/def2‐SVP/6‐31G(d,p), which yielded a deviation of only 0.1%. Notably, the M11/def2‐SVP/def2‐SVP protocol provided satisfactory results for both geometry and activation free energy of the probe complex, highlighting its potential as a balanced choice for structure/reactivity predictions.

Table 5 (right column) shows the 10 best protocols for predicting ${∆G}_{\mathrm{aq}}^{‡}$, all of which showed an absolute deviation lower than 0.52 kcal mol^−1^, which is a fairly good result for such large molecules. For the very best protocol, B3LYP/def2‐SVP/6‐31G(d,p), the relative deviation for structural parameters was only 2.1%, corresponding to a maximum deviation of 0.09 Å for bond lengths and 3.5° for bond angles. This deviation is justified by the fact to use solid‐state structure as reference. Moreover, the B3LYP/def2‐SVP/6‐31G(d) protocol, presented an error of only 0.2% in the calculation of ${∆G}_{\mathrm{aq}}^{‡}$ at a lower computational cost, due to the absence of polarization p‐functions for the hydrogen atom, which can facilitate the study of more complex systems, such as those of biological interest.

### 3.3 Relativistic Effects in the Structure and Reactivity of [Au(dien‐H)Cl]^+^ Complex

**FIGURE 5:**
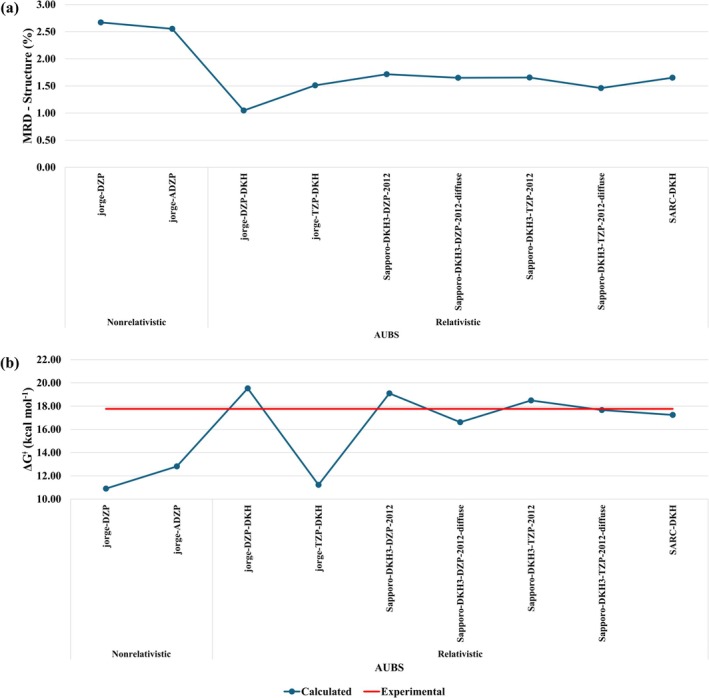
(a) Calculated MRD, Equation (4), for structural parameters. (b) Calculated ${∆G}_{\mathrm{aq}}^{‡}\;\left(\text{kcal}\;\mathrm{mo}l^{-1}\right)$ at nonrelativistic Hamiltonian B3LYP/AUBS/jorge‐DZP/C‐PCM and relativistic Hamiltonian B3LYP‐DKH2/AUBS/jorge‐DZP‐DKH/C‐PCM levels. The red‐line represents the experimental value, ${∆G}_{\mathrm{aq}}^{‡}$ = 17.76 kcal mol^−1^ [25].

Since the relativistic effects strongly influence the description of Au [138], an analysis of the inclusion of the relativistic effects explicitly in the Hamiltonian through the DKH2 approximation was also performed using the ORCA 4.2.1 program [136]. To assess through direct comparison the influence of relativistic effects on the description of the structure and reactivity of the [Au(dien‐H)Cl]^+^ complex, nonrelativistic and scalar relativistic 2nd‐order Douglas‐Kroll‐Hess (DKH2) computational protocols were constructed at B3LYP/ABS/jorge‐DZP and B3LYP‐DKH2/ABS/jorge‐DZP‐DKH levels, where the all‐electron basis sets for the Au atom were used (Tables S9 and S10, Figure 5). It is important to note that when the Hamiltonian with the DKH2 approximation was used, all‐electron basis sets optimized for DKH calculations were also employed. The effects of the solvent were considered using the C‐PCM [135] with the dielectric constant adjusted for water.

A first look at Figure 5a suggests that the inclusion of the relativistic effects in the Hamiltonian contributes to improving the structure. Specifically, the MRD decreased from 2.67% at the B3LYP/jorge‐DZP/jorge‐DZP level to 1.05% at the B3LYP‐DKH2/jorge‐DZP‐DKH/jorge‐DZP‐DKH level. As noted in Table 5, the best geometry obtained from nonrelativistic protocols showed an MRD of 1.42%, indicating that relativistic corrections can further enhance the description of molecular geometry. However, it is important to recognize that the structural parameters of the [Au(dien‐H)Cl]^+^ complex are generally not highly sensitive to the computational protocol. This trend is consistently observed in Figure 5a, where the average MRD for the relativistic protocols was 1.53%, of the same order as nonrelativistic protocols listed in Table 5.

The ${∆G}_{\mathrm{aq}}^{‡}$ was calculated and the values are provided in Table S10. Figure 5b represents the calculated values for all protocols evaluated. The ${∆G}_{\mathrm{aq}}^{‡}$ predicted at nonrelativistic levels were significantly lower than the experimental value, regardless of the basis set for Au, with relative deviations of 38.6% (jorge‐DZP) and 27.9% (jorge‐ADZP). When the relativistic effects are considered by means of the DKH2 approximation, the results are improved, except with jorge‐TZP‐DKH basis set, which provided very small ${∆G}_{\mathrm{aq}}^{‡}$. The best result was obtained with Sapporo‐DKH3‐TZP‐2012‐diffuse (17.66 kcal mol^−1^). Based on the previous results, we conclude that the inclusion of the relativistic effects in the Hamiltonian are important to represent the geometry and reactivity of Au(III) complex, mainly when an all‐electron basis set is used for the Au atom. Therefore, the valence electrons play a primary role, then, the use of the relativistic ECP is enough to obtain a satisfactory description of the kinetic parameters. When necessary, the Hamiltonian relativistic protocol B3LYP‐DKH2/Sapporo‐DKH3‐TZP‐2012‐diffuse/jorge‐DZP‐DKH is recommended since it presented a MRD of 1.46% for structure and 0.6% for activation Gibbs free energy.

**FIGURE 6:**
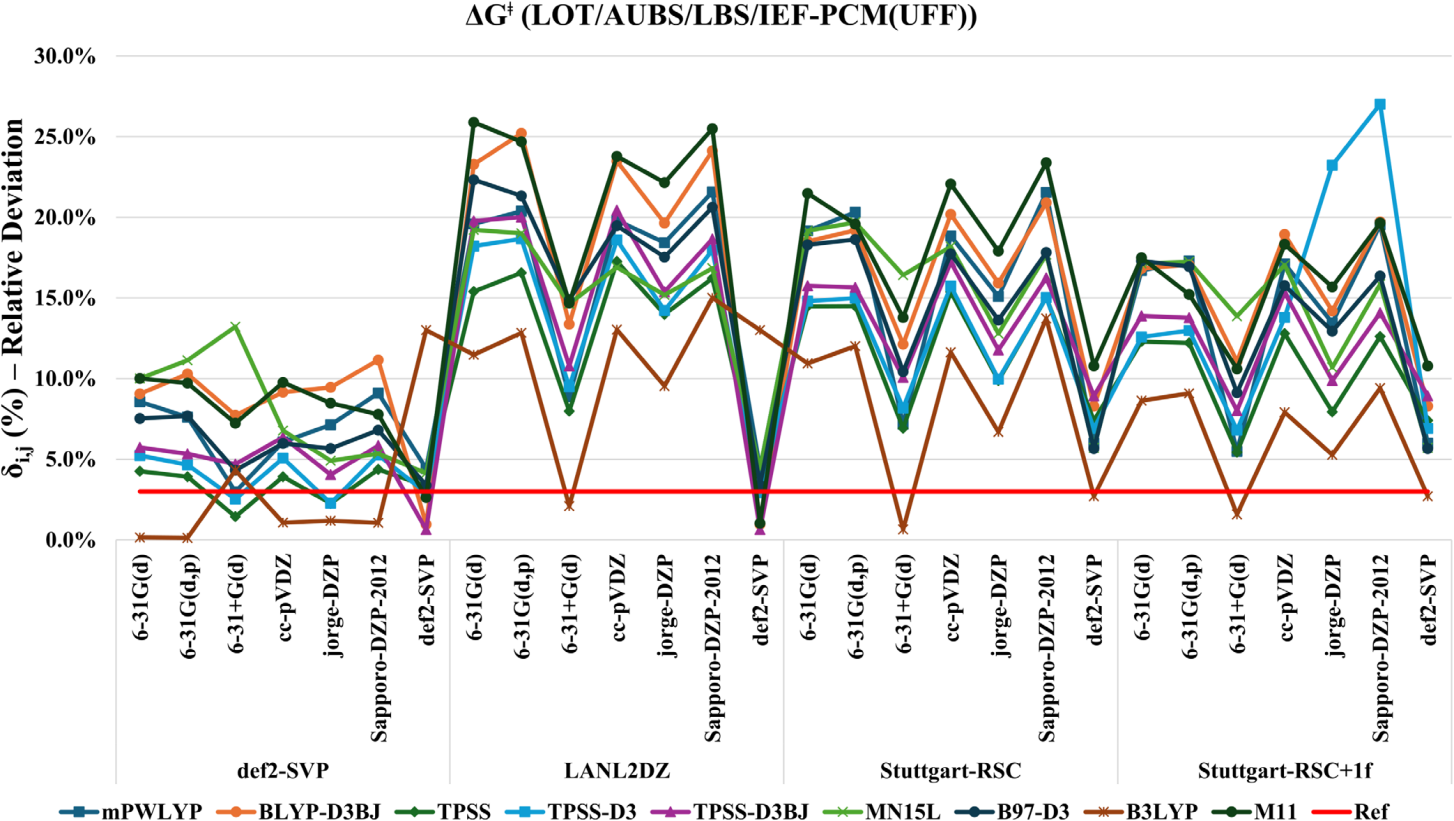
Calculated relative deviation ($\delta_{i,j}$) for ${∆G}_{\mathrm{aq}}^{‡}\;\left(\text{kcal}\;\mathrm{mo}l^{-1}\right)$ with proposed new protocols at LOT/AUBS/LBS levels. The red‐line represents the threshold of 3%, used to select the very best protocols.

Based on the initial benchmarking, new 252 computational protocols were proposed systematically combining AUBS, LBS, and nonrelativistic levels of theory (LOT), that is nonrelativistic Hamiltonians, that individually predicted ${∆G}_{\mathrm{aq}}^{‡}$ with a MRD below 4.5% (i.e., ≤ 1 kcal mol^−1^). The calculated values are presented in Table S11, with corresponding MRD values shown in Figure 6. Notably, the sensitivity of the results to the choice of each protocol component is striking, with MRDs ranging from as low as 0.1% to as high as 27%. Among the tested functionals, B3LYP proved to be the most reliable LOT, featuring in the best‐performing overall protocol (B3LYP/def2‐SVP/6‐31G(d,p)) and in 10 out of the 23 protocols with MRD ≤ 3% (≤ 0.5 kcal mol^−1^). For AUBS, the def2‐SVP basis set delivered the best overall performance, appearing in 14 of the top 23 protocols. Interestingly, this same basis set also performed satisfactorily as LBS, particularly in combination with either LANL2DZ or def2‐SVP AUBS, yielding good results for most DFT functionals, except B3LYP. Specifically, at the B3LYP level, the best AUBS/LBS combination was Stuttgart‐RSC/def2‐SVP. A threshold of 3% MRD (indicated by the red line in Figure 6) was used to filter the most accurate functionals, which were subsequently applied to the test set of molecules.

### 3.4 Prediction of Reactivity of [Au(R‐dien‐H)Cl]^+^ Complexes

Table 6 presents the absolute values of the ${∆G}_{\mathrm{aq}}^{‡}$ calculated for the probe complex and four alkylated derivatives (Figure 1) using the 23 best‐performing nonrelativistic Hamiltonians protocols. For the probe complex (*R* = H), all selected protocols yielded accurate results, with absolute deviations less than 0.5 kcal mol^−1^ (MRD ≤ 3%). As previously discussed, the two best‐performing protocols were B3LYP/def2‐SVP/6‐31G(d,p) and B3LYP/def2‐SVP/6‐31G(d), which showed deviations of less than 0.05 kcal mol^−1^, better than reported in our earlier studies [22, 23].

**TABLE 6:** Calculated ${∆G}_{\mathrm{aq}}^{‡}$ (in kcal mol^−1^) for the aquation reaction of [Au(dien‐H)Cl]^+^ complex and its [Au(R‐dien‐H)Cl]^+^ substituted derivatives at LOT/AUBS/LBS/IEF‐PCM(UFF) levels. The mean absolute deviation (MAD in kcal mol^−1^) and mean relative deviation (MRD in %) are also provided.

	dien‐H	me2	me4	et2	et4	MAD (MRD)
**B3LYP/Stuttgart‐RSC_1f/6‐31 + G(d)**	**18.04**	**17.80**	**19.89**	**19.14**	**20.57**	**0.89 (4.3%)**
B3LYP/def2‐SVP/6‐31G(d)	17.73	15.03	16.49	19.10	16.70	2.54 (12%)
B3LYP/def2‐SVP/6‐31G(d,p)	17.78	14.89	16.33	18.89	16.11	2.76 (13%)
B3LYP/def2‐SVP/cc‐pVDZ	17.95	15.35	17.64	18.78	15.53	2.58 (12%)
B3LYP/def2‐SVP/jorge‐DZP	17.97	14.42	16.75	19.12	17.00	2.58 (13%)
B3LYP/def2‐SVP/Sapporo‐DZP‐2012	17.57	15.05	18.65	18.57	15.43	2.50 (12%)
**B3LYP/LANL2DZ/6‐31 + G(d)**	**17.39**	**18.31**	**17.39**	**18.47**	**20.61**	**1.12 (5.5%)**
**B3LYP/Stuttgart‐RSC/6‐31 + G(d)**	**17.64**	**17.75**	**19.25**	**18.84**	**20.32**	**0.85 (4.0%)**
B3LYP/Stuttgart‐RSC/def2‐SVP	18.24	15.87	18.60	18.74	17.26	1.93 (9.2%)
B3LYP/Stuttgart‐RSC_1f/def2‐SVP	18.53	15.87	19.08	19.39	17.71	1.74 (8.4%)
**TPSS/def2‐SVP/6‐31 + G(d)**	**17.50**	**17.72**	**20.55**	**18.91**	**21.82**	**0.83 (4.2%)**
TPSS/def2‐SVP/jorge‐DZP	17.36	14.24	16.49	18.47	17.73	2.69 (13%)
TPSS‐D3/def2‐SVP/6‐31 + G(d)	17.31	15.13	17.62	17.89	18.26	2.31 (11%)
TPSS‐D3/def2‐SVP/jorge‐DZP	17.36	13.84	16.85	17.56	16.15	3.20 (16%)
TPSS‐D3/def2‐SVP/def2‐SVP	18.28	14.52	17.45	18.59	15.56	2.88 (14%)
TPSS‐D3/LANL2DZ/def2‐SVP	18.28	12.67	14.88	16.72	14.26	4.40 (22%)
mPWLYP/def2‐SVP/6‐31 + G(d)	17.23	17.17	19.00	18.39	19.60	1.27 (6.2%)
TPSS‐D3BJ/def2‐SVP/def2‐SVP	17.87	14.19	17.49	18.66	16.09	2.74 (13%)
TPSS‐D3BJ/LANL2DZ/def2‐SVP	17.87	12.27	14.59	16.78	14.09	4.48 (22%)
BLYP‐D3BJ/def2‐SVP/def2‐SVP	17.93	14.37	16.49	18.11	13.74	3.49 (17%)
BLYP‐D3BJ/LANL2DZ/def2‐SVP	17.93	12.38	13.92	16.08	13.03	4.95 (24%)
M11/def2‐SVP/df2‐SVP	18.23	16.39	19.63	19.17	16.77	1.91 (9.1%)
M11/LANL2DZ/def2‐SVP	17.94	12.66	16.95	16.05	14.94	3.92 (19%)
Experimental [25, 27]	17.76	18.41	19.10	19.69	22.80	—

*Note:* The top four protocols are highlighted in bold.

For the alkylated species, the deviation in ${∆G}_{\mathrm{aq}}^{‡}$ was generally larger than for the reference complex, particularly in cases with bulky substituents (e.g., *R* = Et_4_). For the two previously identified top‐performing protocols, the deviation increased to as much as 6.7 kcal mol^−1^, with an average deviation of 2.5 kcal mol^−1^ over the entire series (~12%). This result suggests that computational protocols optimized for the simplest Au(III) complex may not be directly applicable to more sterically demanding derivatives. As shown in Table 6, all protocols with an average absolute deviation of approximately 1 kcal mol^−1^ employed the 6‐31+G(d) basis set for ligands (LBS). Among these, three used B3LYP as the level of theory (LOT), and two used the Stuttgart‐RSC effective core potential (ECP) as the basis set for gold (AUBS). In contrast, protocols incorporating D3(BJ) dispersion corrections significantly underestimated ${∆G}_{\mathrm{aq}}^{‡}$, with deviations reaching up to 10 kcal mol^−1^ (43%).

In summary, the most accurate and transferable protocol for modeling Au(III) complexes, particularly those with bulky substituents, employs B3LYP as the LOT, the Stuttgart‐RSC ECP for Au (AUBS), and 6‐31+G(d) for ligand atoms (LBS), namely, B3LYP/Stuttgart‐RSC/6‐31+G(d)/IEF‐PCM(UFF), that presented a MRD of only 4.0% considering all five Au(III) complexes studied.

## 4 Concluding Remarks

The choice of computational protocol to calculate the structure and, mainly, kinetic parameters for Au complexes is a challenging and sensitive task. In the present study, we attempt to benchmark these properties using [Au(dien‐H)Cl]^+^ as the probe complex and four of its derivatives as a test set. A total of 397 combinations of DFT‐functional and basis sets were tested, including all features available, such as different types of functionals, ECP and all‐electrons basis set for Au, more than 50 basis sets for ligand atoms, empirical dispersion correction, and relativistic effects.

Among the level of theory tested, we got satisfactory results with mPWLYP, BLYP‐D3BJ, TPSS, TPSS‐D3, TPSS‐D3BJ, MN15L, B97‐D3, B3LYP, and M11 (LC) DFT functionals when used with different combinations of basis sets. For instance, B3LYP/def2‐SVP/6‐31G(d) and BLYP‐D3BJ/def2‐SVP/def2‐SVP provided activation free energy with relative error lower than 1% (~0.2 kcal mol^−1^) when compared to the experimental reference value. However, care must be taken when transferring those protocols to analogue molecules with bulky ligands. We observed that to improve transferability, the basis sets for the metal and ligands should be slightly improved with the addition of sets of diffuse functions, d for Au and s/p for light atoms. Then, Stuttgart‐RSC, which has four sets of d‐functions for Au, is recommended for Au, and the 6‐31+G(d), with one set of diffuse s/p‐functions, is recommended for ligand atoms. B3LYP is suggested as the level of theory for the whole series tested.

## Conflicts of Interest

The authors declare no conflicts of interest.

## Supporting information


Table S1.

## Data Availability

The data that support the findings of this study are available from the corresponding author upon reasonable request.
