# Supplementary material for: A DFT‐Based Protocol for Modeling the Structure and Reactivity of Gold(III) Complexes
Source: J Comput Chem. 2025 Jul 9;46(19):e70179. doi: 10.1002/jcc.70179 (PMC12242115; doi:10.1002/jcc.70179)
Supplement: Supplementary file 1 — Table S1. [file JCC-46-0-s001.docx]

**A DFT-based protocol for modeling the structure and reactivity of gold(III) complexes**

Luana P. P. Cunha^1^, Larissa P. N. Machado^1^, Willian T. G. Novato^1^, Hélio F. Dos Santos^2^, Diego F. S. Paschoal^1,*^

^1^NQTCM: Núcleo de Química Teórica e Computacional de Macaé, Polo Ajuda, Universidade Federal do Rio de Janeiro, Campus UFRJ-Macaé, 27.971-525, Macaé, RJ, Brazil.

^2^NEQC: Núcleo de Estudos em Química Computacional, Departamento de Química - ICE, Universidade Federal de Juiz de Fora, Campus Universitário, 36.036-900, Juiz de Fora, MG, Brazil.

**Supplementary Material**

____________________

*Corresponding author:

Diego F. S. Paschoal, e-mail: [diegopaschoal01@gmail.com](mailto:diegopaschoal01@gmail.com) or [diegofspaschoal@macae.ufrj.br](mailto:diegofspaschoal@macae.ufrj.br), Tel.: +55 (22) 2141- 4015.

**Table S1.** Calculated $Au-N1$, $Au-N4$, and $Au-Cl$ bond lengths (in $Å$) and $N1-Au-N7$, $N1-Au-N4$, $N1-Au-Cl$, and $N4-Au-Cl$ bond angles (in degree) at B3LYP/**AUBS**/def2-SVP/IEF-PCM(UFF) level. The MRD (in %), eq. (4), is also presented.

|  | **AUBS** | **Au-N1** | **Au-N4** | **Au-Cl** | **N1-Au-N7** | **N1-Au-N4** | **N1-Au-Cl** | **N4-Au-Cl** | **MRD** |
| --- | --- | --- | --- | --- | --- | --- | --- | --- | --- |
| ECP | cc-pVDZ-PP | 2.088 | 2.019 | 2.387 | 165.12 | 83.25 | 97.01 | 174.48 | 2.08 |
|  | cc-pVTZ-PP | 2.079 | 2.011 | 2.378 | 165.81 | 83.57 | 96.65 | 175.29 | 1.79 |
|  | cc-pVQZ-PP | 2.074 | 2.007 | 2.373 | 166.28 | 83.76 | 96.44 | 175.61 | 1.62 |
|  | cc-pV5Z-PP | 2.073 | 2.006 | 2.366 | 166.43 | 83.83 | 96.37 | 175.76 | 1.53 |
|  | cc-pwCVDZ-PP | 2.084 | 2.016 | 2.384 | 165.28 | 83.32 | 96.93 | 174.64 | 2.00 |
|  | cc-pwCVTZ-PP | 2.075 | 2.008 | 2.373 | 165.98 | 83.64 | 96.58 | 175.35 | 1.71 |
|  | cc-pwCVQZ-PP | 2.071 | 2.005 | 2.368 | 166.36 | 83.79 | 96.41 | 175.61 | 1.56 |
|  | cc-pwCV5Z-PP | 2.069 | 2.004 | 2.364 | 166.54 | 83.88 | 96.32 | 175.76 | 1.48 |
|  | aug-cc-pVDZ-PP | 2.080 | 2.012 | 2.383 | 166.08 | 83.67 | 96.53 | 175.78 | 1.72 |
|  | aug-cc-pVTZ-PP | 2.074 | 2.006 | 2.376 | 166.44 | 83.85 | 96.35 | 175.52 | 1.61 |
|  | aug-cc-pVQZ-PP | 2.072 | 2.006 | 2.371 | 166.60 | 83.91 | 96.28 | 175.74 | 1.51 |
|  | aug-cc-pV5Z-PP | 2.071 | 2.005 | 2.369 | 166.58 | 83.90 | 96.29 | 175.76 | 1.51 |
|  | CRENBL | 2.093 | 2.030 | 2.387 | 164.79 | 83.05 | 97.18 | 175.32 | 2.07 |
|  | def2-SV | 2.117 | 2.047 | 2.409 | 163.73 | 82.56 | 97.72 | 174.37 | 2.58 |
|  | def2-SVP | 2.097 | 2.026 | 2.395 | 164.94 | 83.11 | 97.14 | 174.55 | 2.20 |
|  | def2-SVPD | 2.098 | 2.030 | 2.398 | 164.83 | 83.08 | 97.18 | 174.52 | 2.22 |
|  | def2-TZVP | 2.086 | 2.019 | 2.376 | 165.39 | 83.35 | 96.88 | 175.09 | 1.89 |
|  | def2-TZVPP | 2.080 | 2.013 | 2.372 | 165.77 | 83.54 | 96.69 | 175.20 | 1.76 |
|  | def2-TZVPD | 2.086 | 2.019 | 2.381 | 165.31 | 83.34 | 96.90 | 174.92 | 1.95 |
|  | def2-TZVPPD | 2.081 | 2.014 | 2.375 | 165.70 | 83.51 | 96.72 | 175.12 | 1.81 |
|  | def2-QZVP | 2.079 | 2.013 | 2.373 | 165.93 | 83.62 | 96.60 | 175.24 | 1.73 |
|  | def2-QZVPP | 2.076 | 2.010 | 2.370 | 166.18 | 83.74 | 96.48 | 175.53 | 1.62 |
|  | LANL2DZ | 2.117 | 2.048 | 2.429 | 163.74 | 82.62 | 97.68 | 174.31 | 2.68 |
|  | modified LANL2DZ | 2.115 | 2.046 | 2.419 | 163.74 | 82.65 | 97.66 | 174.13 | 2.62 |
|  | LANL2TZ | 2.097 | 2.029 | 2.402 | 164.49 | 82.94 | 97.34 | 174.34 | 2.34 |
|  | LANL2TZ(f) | 2.081 | 2.012 | 2.392 | 165.39 | 83.35 | 96.91 | 174.43 | 2.06 |
|  | SBKJC-VDZ | 2.111 | 2.042 | 2.399 | 163.85 | 82.68 | 97.62 | 174.25 | 2.47 |
|  | Stuttgart-RSC | 2.122 | 2.055 | 2.426 | 164.00 | 82.66 | 97.56 | 175.58 | 2.50 |
|  | Stuttgart-RSC+1f | 2.122 | 2.055 | 2.426 | 164.00 | 82.67 | 97.56 | 175.58 | 2.50 |
| ABS | jorge-DZP | 2.147 | 2.052 | 2.418 | 163.08 | 82.33 | 97.97 | 174.80 | 2.90 |
|  | jorge-ADZP | 2.136 | 2.041 | 2.407 | 163.90 | 82.72 | 97.51 | 176.07 | 2.53 |
|  | **Experimental** [1] | **2.031** | **2.060** | **2.333** | **167.62** | **85.41** | **94.79** | **176.43** |  |

**Table S2.** $Au-N1$, $Au-N4$, and $Au-Cl$ bond lengths (in $Å$) and $N1-Au-N7$, $N1-Au-N4$, $N1-Au-Cl$, and $N4-Au-Cl$ bond angles (in degree) at B3LYP/def2-SVP/**LBS**/IEF-PCM(UFF) level. The MRD (in %), eq. (4), is also presented.

|  | **LBS** | **Au-N1** | **Au-N4** | **Au-Cl** | **N1-Au-N7** | **N1-Au-N4** | **N1-Au-Cl** | **N4-Au-Cl** | **MRD** |
| --- | --- | --- | --- | --- | --- | --- | --- | --- | --- |
| Jorge | jorge-DZP | 2.091 | 2.026 | 2.420 | 165.49 | 83.37 | 96.86 | 175.04 | 2.11 |
|  | jorge-TZP | 2.091 | 2.024 | 2.406 | 165.40 | 83.31 | 96.91 | 175.25 | 2.04 |
|  | jorge-QZP | 2.089 | 2.022 | 2.403 | 165.57 | 83.39 | 96.83 | 175.33 | 1.97 |
|  | jorge-ADZP | 2.090 | 2.024 | 2.424 | 165.72 | 83.45 | 96.76 | 175.43 | 2.06 |
|  | jorge-ATZP | 2.090 | 2.024 | 2.405 | 165.44 | 83.34 | 96.88 | 175.32 | 2.01 |
|  | jorge-AQZP | 2.087 | 2.022 | 2.400 | 165.64 | 83.42 | 96.80 | 175.31 | 1.93 |
| Dunning | cc-pVDZ | 2.096 | 2.030 | 2.410 | 165.11 | 83.21 | 97.04 | 174.83 | 2.16 |
|  | cc-pVQZ | 2.091 | 2.023 | 2.405 | 165.55 | 83.38 | 96.84 | 175.19 | 2.01 |
|  | cc-pVTZ | 2.089 | 2.022 | 2.401 | 165.56 | 83.39 | 96.82 | 175.29 | 1.96 |
|  | aug-cc-pVDZ | 2.094 | 2.027 | 2.423 | 165.41 | 83.33 | 96.89 | 175.43 | 2.12 |
|  | aug-cc-pVQZ | 2.089 | 2.024 | 2.406 | 165.56 | 83.39 | 96.83 | 175.33 | 1.98 |
|  | aug-cc-pVTZ | 2.087 | 2.022 | 2.399 | 165.62 | 83.42 | 96.80 | 175.32 | 1.92 |
| Alhrichs | def2-SVP | 2.097 | 2.026 | 2.395 | 164.94 | 83.11 | 97.15 | 174.54 | 2.16 |
|  | def2-SVPD | 2.094 | 2.024 | 2.429 | 165.54 | 83.37 | 96.84 | 175.47 | 2.15 |
|  | def2-TZVP | 2.091 | 2.025 | 2.400 | 165.50 | 83.36 | 96.86 | 175.26 | 1.97 |
|  | def2-TZVPD | 2.091 | 2.025 | 2.404 | 165.46 | 83.35 | 96.87 | 175.33 | 2.00 |
|  | def2-QZVP | 2.089 | 2.023 | 2.400 | 165.53 | 83.39 | 96.83 | 175.32 | 1.95 |
|  | def2-QZVPD | 2.090 | 2.023 | 2.400 | 165.52 | 83.38 | 96.83 | 175.36 | 1.96 |
| Pople | 6-31G | 2.103 | 2.028 | 2.463 | 165.78 | 83.51 | 96.71 | 174.61 | 2.41 |
|  | 6-31+G | 2.103 | 2.028 | 2.470 | 165.86 | 83.53 | 96.68 | 174.76 | 2.42 |
|  | 6-31++G | 2.102 | 2.028 | 2.470 | 165.87 | 83.54 | 96.67 | 174.69 | 2.42 |
|  | 6-31G(d) | 2.096 | 2.027 | 2.418 | 165.35 | 83.31 | 96.92 | 175.00 | 2.15 |
|  | 6-31+G(d) | 2.096 | 2.028 | 2.425 | 165.38 | 83.31 | 96.92 | 175.10 | 2.18 |
|  | 6-31++G(d) | 2.096 | 2.028 | 2.425 | 165.39 | 83.31 | 96.91 | 175.07 | 2.18 |
|  | 6-31G(d,p) | 2.097 | 2.027 | 2.418 | 165.37 | 83.32 | 96.91 | 174.98 | 2.15 |
|  | 6-31+G(d,p) | 2.097 | 2.028 | 2.425 | 165.42 | 83.32 | 96.90 | 175.07 | 2.18 |
|  | 6-31++G(d,p) | 2.096 | 2.028 | 2.425 | 165.44 | 83.33 | 96.90 | 175.04 | 2.18 |
|  | 6-31+G(2d) | 2.092 | 2.027 | 2.415 | 165.47 | 83.35 | 96.87 | 175.35 | 2.06 |
|  | 6-31+G(2d,p) | 2.093 | 2.027 | 2.416 | 165.58 | 83.40 | 96.82 | 175.29 | 2.05 |
|  | 6-31+G(2df) | 2.092 | 2.024 | 2.404 | 165.55 | 83.38 | 96.83 | 175.35 | 1.99 |
|  | 6-31+G(2df,p) | 2.091 | 2.023 | 2.405 | 165.58 | 83.39 | 96.82 | 175.30 | 1.99 |
|  | 6-31+G(2df,2p) | 2.091 | 2.023 | 2.405 | 165.61 | 83.40 | 96.81 | 175.31 | 1.98 |
|  | 6-31++G(2df,2pd) | 2.090 | 2.023 | 2.405 | 165.61 | 83.39 | 96.82 | 175.33 | 1.98 |
|  | 6-311G | 2.103 | 2.027 | 2.478 | 165.74 | 83.44 | 96.75 | 174.95 | 2.49 |
|  | 6-311+G | 2.101 | 2.028 | 2.473 | 165.77 | 83.48 | 96.73 | 174.91 | 2.43 |
|  | 6-311++G | 2.101 | 2.028 | 2.473 | 165.80 | 83.49 | 96.71 | 174.92 | 2.42 |
|  | 6-311G(d) | 2.098 | 2.027 | 2.431 | 165.19 | 83.19 | 97.03 | 175.14 | 2.29 |
|  | 6-311+G(d) | 2.097 | 2.029 | 2.426 | 165.18 | 83.20 | 97.02 | 175.15 | 2.23 |
|  | 6-311++G(d) | 2.097 | 2.029 | 2.426 | 165.18 | 83.20 | 97.02 | 175.15 | 2.23 |
|  | 6-311G(d,p) | 2.099 | 2.027 | 2.431 | 165.22 | 83.20 | 97.02 | 175.10 | 2.30 |
|  | 6-311+G(d,p) | 2.098 | 2.029 | 2.426 | 165.19 | 83.20 | 97.02 | 175.14 | 2.25 |
|  | 6-311++G(d,p) | 2.098 | 2.029 | 2.426 | 165.22 | 83.21 | 97.01 | 175.18 | 2.24 |
|  | 6-311+G(2d) | 2.093 | 2.028 | 2.418 | 165.31 | 83.27 | 96.95 | 175.36 | 2.10 |
|  | 6-311+G(2d,p) | 2.094 | 2.027 | 2.419 | 165.51 | 83.38 | 96.84 | 175.34 | 2.08 |
|  | 6-311+G(2df) | 2.093 | 2.024 | 2.406 | 165.30 | 83.25 | 96.96 | 175.35 | 2.07 |
|  | 6-311+G(2df,p) | 2.093 | 2.024 | 2.407 | 165.43 | 83.32 | 96.89 | 175.34 | 2.05 |
|  | 6-311+G(2df,2p) | 2.093 | 2.024 | 2.407 | 165.44 | 83.32 | 96.89 | 175.37 | 2.04 |
|  | 6-311++G(2df,2pd) | 2.092 | 2.024 | 2.407 | 165.49 | 83.34 | 96.86 | 175.42 | 2.02 |
| Sapporo | DZP-2012 | 2.096 | 2.029 | 2.411 | 165.11 | 83.20 | 97.04 | 174.98 | 2.16 |
|  | DZP-2012-diffuse | 2.096 | 2.028 | 2.413 | 165.23 | 83.26 | 96.97 | 175.32 | 2.11 |
|  | TZP-2012 | 2.089 | 2.023 | 2.400 | 165.55 | 83.39 | 96.83 | 175.19 | 1.96 |
|  | TZP-2012-diffuse | 2.090 | 2.024 | 2.402 | 165.49 | 83.37 | 96.85 | 175.33 | 1.98 |
|  | **Experimental** [1] | **2.053** | **2.060** | **2.333** | **167.62** | **86.81** | **95.45** | **176.43** |  |

**Table S3.** $Au-N1$, $Au-N4$, and $Au-Cl$ bond lengths (in $Å$) and $N1-Au-N7$, $N1-Au-N4$, $N1-Au-Cl$ and, $N4-Au-Cl$ bond angles (in degree) at **LOT**/def2-SVP/def2-SVP/IEF-PCM(UFF) level. The MRD (in %), eq. (4), is also presented.

|  | **LOT** | **Au-N1** | **Au-N4** | **Au-Cl** | **N1-Au-N7** | **N1-Au-N4** | **N1-Au-Cl** | **N4-Au-Cl** | **MRD** |
| --- | --- | --- | --- | --- | --- | --- | --- | --- | --- |
| *ab initio* | HF | 2.068 | 1.964 | 2.403 | 166.16 | 83.46 | 96.69 | 175.75 | 2.12 |
|  | MP2 | 2.054 | 2.002 | 2.354 | 166.19 | 83.67 | 96.55 | 174.85 | 1.47 |
| LDA | SVWN | 2.050 | 1.991 | 2.329 | 165.97 | 83.73 | 96.54 | 174.48 | 1.49 |
| GGA | mPWLYP | 2.129 | 2.064 | 2.414 | 163.83 | 82.76 | 97.59 | 173.89 | 2.57 |
|  | mPWPBE | 2.098 | 2.034 | 2.386 | 164.96 | 83.21 | 97.08 | 174.21 | 2.06 |
|  | mPWPW91 | 2.101 | 2.037 | 2.388 | 164.84 | 83.16 | 97.14 | 174.20 | 2.10 |
|  | OLYP | 2.106 | 2.034 | 2.395 | 164.62 | 82.98 | 97.30 | 174.31 | 2.27 |
|  | BLYP | 2.131 | 2.066 | 2.419 | 163.84 | 82.75 | 97.59 | 173.94 | 2.63 |
|  | XLYP | 2.019 | 1.964 | 2.292 | 166.27 | 83.90 | 96.35 | 174.57 | 2.04 |
|  | BPW91 | 2.103 | 2.038 | 2.393 | 164.87 | 83.16 | 97.13 | 174.25 | 2.13 |
|  | BP86 | 2.102 | 2.039 | 2.391 | 164.90 | 83.20 | 97.10 | 174.21 | 2.09 |
|  | PBE | 2.100 | 2.035 | 2.384 | 164.86 | 83.18 | 97.11 | 174.27 | 2.07 |
|  | SOGGA11 | 2.084 | 2.009 | 2.381 | 166.20 | 83.65 | 96.44 | 175.67 | 1.71 |
| meta-GGA | τ-HCTH | 2.110 | 2.033 | 2.392 | 164.09 | 82.74 | 97.54 | 174.46 | 2.39 |
|  | VSXC | 2.120 | 2.049 | 2.410 | 164.43 | 83.39 | 96.74 | 176.47 | 2.05 |
|  | B97-2 | 2.080 | 2.007 | 2.379 | 165.43 | 83.29 | 96.94 | 174.74 | 1.97 |
|  | BB95 | 2.104 | 2.038 | 2.390 | 164.76 | 83.10 | 97.19 | 174.24 | 2.15 |
|  | TPSS | 2.096 | 2.032 | 2.391 | 165.32 | 83.31 | 96.96 | 174.35 | 2.02 |
|  | M06L | 2.114 | 2.038 | 2.410 | 163.54 | 82.53 | 97.78 | 174.29 | 2.62 |
|  | M11L | 2.083 | 2.006 | 2.387 | 163.87 | 82.55 | 97.72 | 174.35 | 2.44 |
|  | MN12L | 2.047 | 1.983 | 2.368 | 166.88 | 83.97 | 96.24 | 174.73 | 1.57 |
|  | MN15L | 2.082 | 2.016 | 2.389 | 165.78 | 83.58 | 96.65 | 175.02 | 1.84 |
| Hybrid | mPW1LYP | 2.094 | 2.022 | 2.393 | 164.99 | 83.13 | 97.12 | 174.61 | 2.14 |
|  | mPW1PW91 | 2.070 | 1.998 | 2.369 | 165.79 | 83.46 | 96.77 | 174.83 | 1.80 |
|  | mPW3PBE | 2.074 | 2.004 | 2.371 | 165.67 | 83.43 | 96.81 | 174.70 | 1.83 |
|  | O3LYP | 2.090 | 2.017 | 2.385 | 164.97 | 83.12 | 97.14 | 174.52 | 2.12 |
|  | B3LYP | 2.097 | 2.026 | 2.395 | 164.94 | 83.11 | 97.14 | 174.55 | 2.16 |
|  | X3LYP | 2.094 | 2.023 | 2.391 | 165.01 | 83.15 | 97.11 | 174.59 | 2.12 |
|  | B3PW91 | 2.077 | 2.007 | 2.376 | 165.59 | 83.39 | 96.85 | 174.71 | 1.89 |
|  | B3P86 | 2.073 | 2.004 | 2.370 | 165.65 | 83.42 | 96.82 | 174.75 | 1.82 |
|  | PBE0 | 2.068 | 1.996 | 2.366 | 165.85 | 83.50 | 96.72 | 174.88 | 1.76 |
|  | BHandH | 2.029 | 1.957 | 2.326 | 166.57 | 83.81 | 96.37 | 175.41 | 1.73 |
|  | BHandHLYP | 2.069 | 1.988 | 2.377 | 165.73 | 83.37 | 96.84 | 175.20 | 1.92 |
|  | SOGGA11X | 2.076 | 1.997 | 2.378 | 165.45 | 83.30 | 96.93 | 174.80 | 1.99 |
| Hybrid meta-GGA | τ-HCTHhyb | 2.081 | 2.017 | 2.382 | 165.60 | 83.42 | 96.81 | 174.61 | 1.87 |
|  | BMK | 2.059 | 1.990 | 2.396 | 166.43 | 83.75 | 96.46 | 175.10 | 1.77 |
|  | B1B95 | 2.067 | 1.994 | 2.367 | 165.84 | 83.46 | 96.76 | 174.91 | 1.79 |
|  | TPSSh | 2.084 | 2.017 | 2.383 | 165.62 | 83.40 | 96.83 | 174.57 | 1.91 |
|  | PW6B95 | 2.074 | 2.001 | 2.371 | 165.45 | 83.31 | 96.92 | 174.80 | 1.90 |
|  | M06-HF | 2.028 | 1.950 | 2.354 | 168.52 | 84.63 | 95.49 | 176.17 | 1.53 |
|  | M06 | 2.097 | 2.018 | 2.384 | 164.33 | 82.92 | 97.36 | 174.67 | 2.25 |
|  | M06-2X | 2.079 | 1.986 | 2.396 | 166.01 | 83.57 | 96.64 | 175.22 | 2.02 |
|  | MN15 | 2.056 | 1.986 | 2.350 | 166.69 | 83.90 | 96.30 | 175.20 | 1.42 |
| Long-range corrected | ωB97 | 2.068 | 1.978 | 2.364 | 165.93 | 83.52 | 96.68 | 175.44 | 1.81 |
|  | ωB97xD | 2.074 | 1.990 | 2.375 | 165.56 | 83.37 | 96.86 | 175.13 | 1.95 |
|  | LC-BLYP | 2.038 | 1.955 | 2.333 | 166.61 | 83.82 | 96.36 | 175.65 | 1.61 |
|  | LC-ωPBE | 2.048 | 1.962 | 2.345 | 166.65 | 83.81 | 96.37 | 175.60 | 1.57 |
|  | CAM-B3LYP | 2.070 | 1.992 | 2.368 | 165.74 | 83.43 | 96.79 | 175.01 | 1.84 |
|  | M11 | 2.056 | 1.978 | 2.373 | 167.20 | 84.06 | 96.10 | 175.86 | 1.46 |
|  | MN12SX | 2.054 | 1.988 | 2.370 | 166.54 | 83.80 | 96.42 | 174.82 | 1.59 |
|  | **Experimental** [1] | **2.053** | **2.060** | **2.333** | **167.62** | **86.81** | **95.45** | **176.43** |  |

**Table S4.** $Au-N1$, $Au-N4$, and $Au-Cl$ bond lengths (in $Å$) and $N1-Au-N7$, $N1-Au-N4$, $N1-Au-Cl$, and $N4-Au-Cl$ bond angles (in degree) at **LOT-Dispersion**/def2-SVP/def2-SVP/IEF-PCM(UFF) level. The MRD (in %), eq. (4), is also presented.

|  | **LOT** | **Au-N1** | **Au-N4** | **Au-Cl** | **N1-Au-N7** | **N1-Au-N4** | **N1-Au-Cl** | **N4-Au-Cl** | **MRD** |
| --- | --- | --- | --- | --- | --- | --- | --- | --- | --- |
| D3 | BLYP-D3 | 2.132 | 2.063 | 2.412 | 164.00 | 82.90 | 97.50 | 174.20 | 2.49 |
|  | BP86-D3 | 2.102 | 2.037 | 2.385 | 165.00 | 83.30 | 97.00 | 174.40 | 2.02 |
|  | PBE-D3 | 2.100 | 2.034 | 2.382 | 164.91 | 83.23 | 97.07 | 174.37 | 2.04 |
|  | B97D3 | 2.120 | 2.049 | 2.401 | 164.20 | 82.90 | 97.40 | 174.40 | 2.35 |
|  | TPSS-D3 | 2.096 | 2.030 | 2.388 | 165.41 | 83.39 | 96.88 | 174.50 | 1.96 |
|  | M06-D3 | 2.097 | 2.019 | 2.383 | 164.29 | 82.90 | 97.38 | 174.64 | 2.26 |
|  | B3LYP-D3 | 2.098 | 2.025 | 2.390 | 165.00 | 83.20 | 97.10 | 174.70 | 2.11 |
|  | B3PW91-D3 | 2.078 | 2.005 | 2.371 | 165.70 | 83.50 | 96.80 | 174.90 | 1.82 |
|  | PBE0-D3 | 2.068 | 1.996 | 2.363 | 165.87 | 83.55 | 96.68 | 174.93 | 1.73 |
|  | cam-B3LYP-D3 | 2.071 | 1.992 | 2.365 | 165.78 | 83.50 | 96.73 | 175.06 | 1.80 |
|  | LC-ωPBE-D3 | 2.048 | 1.961 | 2.342 | 166.69 | 83.87 | 96.31 | 175.64 | 1.53 |
| D3BJ | BLYP-D3BJ | 2.122 | 2.059 | 2.411 | 164.28 | 82.98 | 97.34 | 174.32 | 2.33 |
|  | BP86-D3BJ | 2.096 | 2.035 | 2.385 | 165.16 | 83.35 | 96.94 | 174.42 | 1.95 |
|  | PBE-D3BJ | 2.096 | 2.032 | 2.381 | 165.00 | 83.27 | 97.02 | 174.42 | 1.99 |
|  | B97-D3BJ | 2.108 | 2.043 | 2.398 | 164.55 | 83.06 | 97.23 | 174.53 | 2.20 |
|  | TPSS-D3BJ | 2.092 | 2.029 | 2.387 | 165.50 | 83.42 | 96.84 | 174.53 | 1.92 |
|  | B3LYP-D3BJ | 2.091 | 2.022 | 2.389 | 165.20 | 83.30 | 97.00 | 174.80 | 2.02 |
|  | B3PW91-D3BJ | 2.072 | 2.003 | 2.370 | 165.84 | 83.53 | 96.71 | 174.94 | 1.75 |
|  | PBE0-D3BJ | 2.065 | 1.994 | 2.363 | 165.95 | 83.56 | 96.66 | 174.99 | 1.70 |
|  | CAM-B3LYP-D3BJ | 2.068 | 1.991 | 2.366 | 165.83 | 83.49 | 96.73 | 175.10 | 1.79 |
|  | LC-ωPBE-D3BJ | 2.048 | 1.961 | 2.342 | 166.70 | 83.90 | 96.30 | 175.60 | 1.53 |
|  | **Experimental** [1] | **2.053** | **2.060** | **2.333** | **167.62** | **86.81** | **95.45** | **176.43** |  |

**Table S5.** Calculated ${\Delta G}_{\mathrm{aq}}^{\ddagger} (kcal mol^{-1})$ and $k$ (M^-1^ s^-1^) at B3LYP/**AUBS**/def2-SVP/IEF-PCM(UFF) level. The relative deviation, $\delta_{i,j}$(%) – eq. 3, is also presented.

|  | **AUBS** | ${\Delta G}_{\mathrm{aq}}^{\ddagger}$ | $\boldsymbol{\delta}_{\mathbf{i,j}}$ | $k$ | $\boldsymbol{\delta}_{\mathbf{i,j}}$ |
| --- | --- | --- | --- | --- | --- |
| ECP | cc-pVDZ-PP | 20.20 | 13.7% | 1.01x10^-2^ | 98.3% |
|  | cc-pVTZ-PP | 20.26 | 14.1% | 9.10x10^-3^ | 98.5% |
|  | cc-pVQZ-PP | 20.38 | 14.7% | 7.44x10^-3^ | 98.8% |
|  | cc-pV5Z-PP | 20.44 | 15.1% | 6.63x10^-3^ | 98.9% |
|  | cc-pwCVDZ-PP | 20.43 | 15.0% | 6.80x10^-3^ | 98.9% |
|  | cc-pwCVTZ-PP | 20.36 | 14.7% | 7.58x10^-3^ | 98.7% |
|  | cc-pwCVQZ-PP | 20.61 | 16.0% | 5.04x10^-3^ | 99.2% |
|  | cc-pwCV5Z-PP | 20.46 | 15.2% | 6.43x10^-3^ | 98.9% |
|  | aug-cc-pVDZ-PP | 20.31 | 14.3% | 8.34x10^-3^ | 98.6% |
|  | aug-cc-pVTZ-PP | 19.86 | 11.8% | 1.79x10^-2^ | 97.0% |
|  | aug-cc-pVQZ-PP | 19.84 | 11.7% | 1.83x10^-2^ | 97.0% |
|  | aug-cc-pV5Z-PP | 19.91 | 12.1% | 1.64x10^-2^ | 97.3% |
|  | CRENBL | 21.25 | 19.7% | 1.70x10^-3^ | 99.7% |
|  | def2-SV | 19.55 | 10.1% | 2.98x10^-2^ | 95.0% |
|  | def2-SVP | 20.07 | 13.0% | 1.25x10^-2^ | 97.9% |
|  | def2-SVPD | 19.75 | 11.2% | 2.15x10^-2^ | 96.4% |
|  | def2-TZVP | 20.92 | 17.8% | 2.96x10^-3^ | 99.5% |
|  | def2-TZVPP | 20.85 | 17.4% | 3.33x10^-3^ | 99.4% |
|  | def2-TZVPD | 20.50 | 15.4% | 5.99x10^-3^ | 99.0% |
|  | def2-TZVPPD | 20.54 | 15.7% | 5.63x10^-3^ | 99.1% |
|  | def2-QZVP | 20.07 | 13.0% | 1.25x10^-2^ | 97.9% |
|  | def2-QZVPP | 19.93 | 12.2% | 1.58x10^-2^ | 97.4% |
|  | LANL2DZ | 18.34 | 3.3% | 2.28x10^-1^ | 61.9% |
|  | modified LANL2DZ | 18.60 | 4.7% | 1.50x10^-1^ | 75.1% |
|  | LANL2TZ | 19.67 | 10.8% | 2.45x10^-2^ | 95.9% |
|  | LANL2TZ(f) | 20.02 | 12.7% | 1.36x10^-2^ | 97.7% |
|  | SBKJC-VDZ | 19.45 | 9.5% | 3.56x10^-2^ | 94.1% |
|  | Stuttgart-RSC | 18.24 | 2.7% | 2.74x10^-1^ | 54.3% |
|  | Stuttgart-RSC+1f | 18.53 | 4.3% | 1.67x10^-1^ | 72.1% |
| ABS | jorge-DZP | 13.62 | 23.3% | 6.59x10^2^ | >100% |
|  | jorge-ADZP | 15.61 | 12.1% | 2.31x10^1^ | >100% |
|  | **Experimental** [2] | **17.76** | - | **6.00x10^-1^** | - |

**Table S6.** Calculated ${\Delta G}_{\mathrm{aq}}^{\ddagger} (kcal mol^{-1})$ and $k$ (M^-1^ s^-1^) at B3LYP/def2-SVP/**LBS**/IEF-PCM(UFF) level. The relative deviation, $\delta_{i,j}$(%) – eq. 3, is also presented.

|  | **LBS** | ${\Delta G}_{\mathrm{aq}}^{\ddagger}$ | $\boldsymbol{\delta}_{\mathbf{i,j}}$ | $k$ | $\boldsymbol{\delta}_{\mathbf{i,j}}$ |
| --- | --- | --- | --- | --- | --- |
| Jorge | jorge-DZP | 17.97 | 1.2% | 4.26x10^-1^ | 29.0% |
|  | jorge-TZP | 19.91 | 12.1% | 1.62x10^-2^ | 97.3% |
|  | jorge-QZP | 20.20 | 13.8% | 9.94x10^-3^ | 98.3% |
|  | jorge-ADZP | 19.04 | 7.2% | 7.04x10^-2^ | 88.3% |
|  | jorge-ATZP | 20.05 | 12.9% | 1.29x10^-2^ | 97.9% |
|  | jorge-AQZP | 20.25 | 14.0% | 9.14x10^-3^ | 98.5% |
| Dunning | cc-pVDZ | 17.95 | 1.1% | 4.41x10^-1^ | 26.5% |
|  | cc-pVQZ | 19.34 | 8.9% | 4.29x10^-2^ | 92.9% |
|  | cc-pVTZ | 20.03 | 12.8% | 1.34x10^-2^ | 97.8% |
|  | aug-cc-pVDZ | 19.19 | 8.0% | 5.52x10^-2^ | 90.8% |
|  | aug-cc-pVQZ | 19.96 | 12.4% | 1.49x10^-2^ | 97.5% |
|  | aug-cc-pVTZ | 20.32 | 14.4% | 8.14x10^-3^ | 98.6% |
| Alhrichs | def2-SVP | 20.07 | 13.0% | 1.25x10^-2^ | 97.9% |
|  | def2-SVPD | 19.48 | 9.7% | 3.37x10^-2^ | 94.4% |
|  | def2-TZVP | 20.20 | 13.7% | 1.00x10^-2^ | 98.3% |
|  | def2-TZVPD | 20.10 | 13.2% | 1.18x10^-2^ | 98.0% |
|  | def2-QZVP | 20.38 | 14.8% | 7.37x10^-3^ | 98.8% |
|  | def2-QZVPD | 20.32 | 14.4% | 8.19x10^-3^ | 98.6% |
| Pople | 6-31G | 15.80 | 11.1% | 1.68x10^1^ | >100% |
|  | 6-31+G | 16.66 | 6.2% | 3.92x10^0^ | >100% |
|  | 6-31++G | 16.55 | 6.8% | 4.70x10^0^ | >100% |
|  | 6-31G(d) | 17.73 | 0.2% | 6.45x10^-1^ | 7.5% |
|  | 6-31+G(d) | 18.53 | 4.4% | 1.66x10^-1^ | 72.3% |
|  | 6-31++G(d) | 18.47 | 4.0% | 1.85x10^-1^ | 69.2% |
|  | 6-31G(d,p) | 17.78 | 0.1% | 5.94x10^-1^ | 1.0% |
|  | 6-31+G(d,p) | 18.90 | 6.4% | 8.97x10^-2^ | 85.1% |
|  | 6-31++G(d,p) | 18.83 | 6.0% | 1.01x10^-1^ | 83.2% |
|  | 6-31+G(2d) | 18.91 | 6.4% | 8.87x10^-2^ | 85.2% |
|  | 6-31+G(2d,p) | 19.10 | 7.5% | 6.40x10^-2^ | 89.3% |
|  | 6-31+G(2df) | 19.50 | 9.8% | 3.28x10^-2^ | 94.5% |
|  | 6-31+G(2df,p) | 19.71 | 11.0% | 2.30x10^-2^ | 96.2% |
|  | 6-31+G(2df,2p) | 19.81 | 11.5% | 1.93x10^-2^ | 96.8% |
|  | 6-31++G(2df,2pd) | 19.87 | 11.9% | 1.76x10^-2^ | 97.1% |
|  | 6-311G | 15.15 | 14.7% | 4.98x10^1^ | >100% |
|  | 6-311+G | 17.02 | 4.2% | 2.15x10^0^ | >100% |
|  | 6-311++G | 16.97 | 4.4% | 2.32x10^0^ | >100% |
|  | 6-311G(d) | 16.16 | 9.0% | 9.03x10^0^ | >100% |
|  | 6-311+G(d) | 18.42 | 3.7% | 2.03x10^-1^ | 66.2% |
|  | 6-311++G(d) | 18.37 | 3.4% | 2.19x10^-1^ | 63.5% |
|  | 6-311G(d,p) | 17.00 | 4.3% | 2.21x10^0^ | >100% |
|  | 6-311+G(d,p) | 19.19 | 8.0% | 5.50x10^-2^ | 90.8% |
|  | 6-311++G(d,p) | 19.15 | 7.8% | 5.87x10^-2^ | 90.2% |
|  | 6-311+G(2d) | 18.80 | 5.8% | 1.07x10^-1^ | 82.2% |
|  | 6-311+G(2d,p) | 19.37 | 9.1% | 4.04x10^-2^ | 93.3% |
|  | 6-311+G(2df) | 19.56 | 10.1% | 2.93x10^-2^ | 95.1% |
|  | 6-311+G(2df,p) | 19.94 | 12.3% | 1.56x10^-2^ | 97.4% |
|  | 6-311+G(2df,2p) | 19.72 | 11.1% | 2.23x10^-2^ | 96.3% |
|  | 6-311++G(2df,2pd) | 19.71 | 11.0% | 2.29x10^-2^ | 96.2% |
| Sapporo | Sapporo-DZP-2012 | 17.58 | 1.0% | 8.36x10^-1^ | 39.4% |
|  | Sapporo-DZP-2012-diffuse | 19.58 | 10.2% | 2.86x10^-2^ | 95.2% |
|  | Sapporo-TZP-2012 | 19.73 | 11.1% | 2.20x10^-2^ | 96.3% |
|  | Sapporo-TZP-2012-diffuse | 20.10 | 13.2% | 1.19x10^-2^ | 98.0% |
|  | **Experimental** [2] | **17.76** | **-** | **6.00x10^-1^** | **-** |

**Table S7.** Calculated ${\Delta G}_{\mathrm{aq}}^{\ddagger} (kcal mol^{-1})$ and $k$ (s^-1^) at **LOT**/def2-SVP/def2-SVP/IEF-PCM(UFF) level. The relative deviation, $\delta_{i,j}$(%) – eq. 3, is also presented.

|  | **LOT** | ${\Delta G}_{\mathrm{aq}}^{\ddagger}$ | $\boldsymbol{\delta}_{\mathbf{i,j}}$ | $k$ | $\boldsymbol{\delta}_{\mathbf{i,j}}$ |
| --- | --- | --- | --- | --- | --- |
| *ab initio* | HF | 20.96 | 18.0% | 2.80x10^-3^ | 99.5% |
|  | MP2 | 21.52 | 21.2% | 1.08x10^-3^ | 99.8% |
| LDA | SVWN | 19.52 | 9.9% | 3.17x10^-2^ | 94.7% |
| GGA | mPWLYP | 18.54 | 4.4% | 1.64x10^-1^ | 72.7% |
|  | mPWPBE | 19.26 | 8.5% | 4.85x10^-2^ | 91.9% |
|  | mPWPW91 | 19.14 | 7.8% | 5.95x10^-2^ | 90.1% |
|  | OLYP | 21.30 | 19.9% | 1.57x10^-3^ | 99.7% |
|  | BLYP | 18.63 | 4.9% | 1.41x10^-1^ | 76.5% |
|  | XLYP | 19.71 | 11.0% | 2.27x10^-2^ | 96.2% |
|  | BPW91 | 19.34 | 8.9% | 4.24x10^-2^ | 92.9% |
|  | BP86 | 19.08 | 7.4% | 6.62x10^-2^ | 89.0% |
|  | PBE | 19.19 | 8.1% | 5.48x10^-2^ | 90.9% |
|  | SOGGA11 | 21.75 | 22.5% | 7.32x10^-4^ | 99.9% |
| meta-GGA | τ-HCTH | 20.55 | 15.7% | 5.51x10^-3^ | 99.1% |
|  | VSXC | 14.05 | 20.9% | 3.20x10^+2^ | >100% |
|  | B97-2 | 20.66 | 16.3% | 4.63x10^-3^ | 99.2% |
|  | BB95 | 18.33 | 3.2% | 2.35x10^-1^ | 60.9% |
|  | TPSS | 18.35 | 3.3% | 2.26x10^-1^ | 62.4% |
|  | M06L | 16.13 | 9.2% | 9.57x10^0^ | >100% |
|  | M11L | 15.15 | 14.7% | 5.00x10^+1^ | >100% |
|  | MN12L | 19.23 | 8.3% | 5.10x10^-2^ | 91.5% |
|  | MN15L | 18.49 | 4.1% | 1.77x10^-1^ | 70.5% |
| Hybrid | mPW1LYP | 20.02 | 12.7% | 1.35x10^-2^ | 97.8% |
|  | mPW1PW91 | 20.54 | 15.7% | 5.59x10^-3^ | 99.1% |
|  | mPW3PBE | 20.43 | 15.0% | 6.82x10^-3^ | 98.9% |
|  | O3LYP | 21.72 | 22.3% | 7.64x10^-4^ | 99.9% |
|  | B3LYP | 20.07 | 13.0% | 1.25x10^-2^ | 97.9% |
|  | X3LYP | 20.05 | 12.9% | 1.29x10^-2^ | 97.8% |
|  | B3PW91 | 20.47 | 15.2% | 6.37x10^-3^ | 98.9% |
|  | B3P86 | 20.29 | 14.2% | 8.65x10^-3^ | 98.6% |
|  | PBE0 | 20.54 | 15.7% | 5.64x10^-3^ | 99.1% |
|  | BHandH | 20.49 | 15.3% | 6.17x10^-3^ | 99.0% |
|  | BHandHLYP | 20.53 | 15.6% | 5.74x10^-3^ | 99.0% |
|  | SOGGA11X | 20.23 | 13.9% | 9.58x10^-3^ | 98.4% |
| Hybrid meta-GGA | τ-HCTHhyb | 20.17 | 13.6% | 1.05x10^-2^ | 98.3% |
|  | BMK | 20.87 | 17.5% | 3.24x10^-3^ | 99.5% |
|  | B1B95 | 19.68 | 10.8% | 2.38x10^-2^ | 96.0% |
|  | TPSSh | 19.00 | 7.0% | 7.59x10^-2^ | 87.4% |
|  | PW6B95 | 19.09 | 7.5% | 6.53x10^-2^ | 89.1% |
|  | M06-HF | 18.66 | 5.1% | 1.34x10^-1^ | 77.7% |
|  | M06 | 18.86 | 6.2% | 9.50x10^-2^ | 84.2% |
|  | M06-2X | 16.49 | 7.1% | 5.20x10^0^ | >100% |
|  | MN15 | 19.82 | 11.6% | 1.89x10^-2^ | 96.8% |
| Long-range corrected | ωB97 | 18.97 | 6.8% | 7.94x10^-2^ | 86.8% |
|  | ωB97xD | 20.21 | 13.8% | 9.88x10^-3^ | 98.4% |
|  | LC-BLYP | 20.83 | 17.3% | 3.47x10^-3^ | 99.4% |
|  | LC-ωPBE | 20.97 | 18.1% | 2.71x10^-3^ | 99.5% |
|  | CAM-B3LYP | 20.12 | 13.3% | 1.14x10^-2^ | 98.1% |
|  | M11 | 18.23 | 2.63% | 2.79x10^-1^ | 53.5% |
|  | MN12SX | 19.79 | 11.4% | 2.01x10^-2^ | 96.7% |
|  | **Experimental** [2] | **17.76** | **-** | **6.00x10^-1^** | **-** |

**Table S8.** Calculated ${\Delta G}_{\mathrm{aq}}^{\ddagger} (kcal mol^{-1})$ and $k$ (s^-1^) at **LOT-Dispersion**/def2-SVP/def2-SVP/IEF-PCM(UFF) level. The relative deviation, $\delta_{i,j}$(%) – eq. 3, is also presented.

|  | **LOT** | ${\Delta G}_{\mathrm{aq}}^{\ddagger}$ | $\boldsymbol{\delta}_{\mathbf{i,j}}$ | $k$ | $\boldsymbol{\delta}_{\mathbf{i,j}}$ |
| --- | --- | --- | --- | --- | --- |
| D3 | BLYP-D3 | 18.90 | 6.4% | 9.02x10^-2^ | 85.0% |
|  | BP86-D3 | 19.18 | 8.0% | 5.58x10^-2^ | 90.7% |
|  | PBE-D3 | 19.20 | 8.1% | 5.36x10^-2^ | 91.1% |
|  | B97-D3 | 18.36 | 3.4% | 2.22x10^-1^ | 63.0% |
|  | TPSS-D3 | 18.28 | 2.92% | 2.55x10^-1^ | 57.4% |
|  | M06-D3 | 18.75 | 5.6% | 1.15x10^-1^ | 80.9% |
|  | B3LYP-D3 | 19.62 | 10.5% | 2.66x10^-2^ | 95.6% |
|  | B3PW91-D3 | 20.38 | 14.7% | 7.43x10^-3^ | 98.8% |
|  | PBE0-D3 | 20.31 | 14.4% | 8.31x10^-3^ | 98.6% |
|  | CAM-B3LYP-D3 | 19.75 | 11.2% | 2.14x10^-2^ | 96.4% |
|  | LC-wPBE-D3 | 20.63 | 16.1% | 4.87x10^-3^ | 99.2% |
| D3BJ | BLYP-D3BJ | 17.93 | 0.97% | 4.58x10^-1^ | 23.6% |
|  | BP86-D3BJ | 18.79 | 5.8% | 1.09x10^-1^ | 81.9% |
|  | PBE-D3BJ | 18.87 | 6.2% | 9.43x10^-2^ | 84.3% |
|  | B97-D3BJ | 18.81 | 5.9% | 1.05x10^-1^ | 82.5% |
|  | TPSS-D3BJ | 17.87 | 0.64% | 5.05x10^-1^ | 15.8% |
|  | B3LYP-D3BJ | 19.23 | 8.3% | 5.11x10^-2^ | 91.5% |
|  | B3PW91-D3BJ | 19.81 | 11.5% | 1.94x10^-2^ | 96.8% |
|  | PBE0-D3BJ | 20.23 | 13.9% | 9.57x10^-3^ | 98.4% |
|  | CAM-B3LYP-D3BJ | 19.72 | 11.0% | 2.25x10^-2^ | 96.3% |
|  | LC-wPBE-D3BJ | 20.24 | 14.0% | 9.29x10^-3^ | 98.5% |
|  | **Experimental** [2] | **17.76** | **-** | **6.00x10^-1^** | **-** |

**Table S9.** $Au-N1$, $Au-N4$ and $Au-Cl$ bond lengths (in $Å$) and $N1-Au-N7$, $N1-Au-N4$, $N1-Au-Cl$, and $N4-Au-Cl$ bond angles (in degree) at B3LYP/**AUBS**/jorge-DZP/C-PCM and B3LYP-DKH2/**AUBS**/jorge-DZP-DKH/C-PCM levels. The MRD (in %), eq. (4), is also presented.

|  | **AUBS** | **Au-N1** | **Au-N4** | **Au-Cl** | **N1-Au-N7** | **N1-Au-N4** | **N1-Au-Cl** | **N4-Au-Cl** | **MRD** |
| --- | --- | --- | --- | --- | --- | --- | --- | --- | --- |
| NR | jorge-DZP | 2.136 | 2.051 | 2.444 | 163.9 | 82.7 | 97.4 | 176.1 | 2.67 |
|  | jorge-ADZP | 2.130 | 2.047 | 2.436 | 164.1 | 82.8 | 97.3 | 176.7 | 2.55 |
| REL | jorge-DZP-DKH | 2.032 | 1.985 | 2.374 | 168.2 | 84.8 | 95.4 | 176.1 | 1.05 |
|  | jorge-TZP-DKH | 2.062 | 1.973 | 2.404 | 168.2 | 84.8 | 95.4 | 176.3 | 1.51 |
|  | Sapporo-DKH3-DZP-2012 | 2.065 | 2.007 | 2.405 | 166.3 | 83.8 | 96.4 | 175.9 | 1.71 |
|  | Sapporo-DKH3-DZP-2012-diffuse | 2.056 | 2.002 | 2.404 | 166.3 | 84.0 | 96.2 | 175.5 | 1.65 |
|  | Sapporo-DKH3-TZP-2012 | 2.059 | 1.998 | 2.400 | 166.6 | 84.0 | 96.3 | 175.6 | 1.65 |
|  | Sapporo-DKH3-TZP-2012-diffuse | 2.044 | 1.989 | 2.395 | 166.9 | 84.3 | 96.0 | 175.6 | 1.46 |
|  | SARC-DKH | 2.055 | 1.998 | 2.400 | 166.6 | 83.9 | 96.3 | 175.5 | 1.65 |
|  | **Experimental** [1] | **2.031** | **2.060** | **2.333** | **167.6** | **85.4** | **94.8** | **176.4** |  |

NR = nonrelativistic Hamiltonian / REL = relativistic Hamiltonian.

**Table S10.** Calculated ${\Delta G}_{\mathrm{aq}}^{\ddagger} (kcal mol^{-1})$ and $k$ (s^-1^) at B3LYP/**AUBS**/jorge-DZP/C-PCM and B3LYP-DKH2/**AUBS**/jorge-DZP-DKH/C-PCM levels. The relative deviation, $\delta_{i,j}$(%) – eq. 3, is also presented.

|  | **AUBS** | ${\Delta G}_{\mathrm{aq}}^{\ddagger}$ | $\boldsymbol{\delta}_{\mathbf{i,j}}$ | $k$ | $\boldsymbol{\delta}_{\mathbf{i,j}}$ |
| --- | --- | --- | --- | --- | --- |
| NR | jorge-DZP | 10.90 | 38.6% | 6.47x10^4^ | >100% |
|  | jorge-ADZP | 12.81 | 27.9% | 2.57x10^3^ | >100% |
| REL | jorge-DZP-DKH | 19.52 | 56.7% | 3.12x10^-2^ | 94.8% |
|  | jorge-TZP-DKH | 11.22 | 44.4% | 3.78x10^4^ | >100% |
|  | Sapporo-DKH3-DZP-2012 | 19.10 | 9.9% | 6.41x10^-2^ | 89.3% |
|  | Sapporo-DKH3-DZP-2012-diffuse | 16.61 | 36.8% | 4.24x10^0^ | >100% |
|  | Sapporo-DKH3-TZP-2012 | 18.49 | 7.5% | 1.79x10^-1^ | 70.2% |
|  | Sapporo-DKH3-TZP-2012-diffuse | 17.66 | 6.5% | 7.29x10^-1^ | 21.5% |
|  | SARC-DKH | 17.23 | 4.1% | 1.49x10^0^ | >100% |
|  | **Experimental** [2] | **17.76** | **-** | **6.00x10^-1^** | **-** |

NR = nonrelativistic Hamiltonian / REL = relativistic Hamiltonian.

**Table S11.** Calculated ${\Delta G}_{\mathrm{aq}}^{\ddagger} (kcal mol^{-1})$ at LOT/AUBS/LBS/IEF-PCM(UFF) levels.

| **AUBS** | **LBS** | **mPWLYP** | **BLYP-D3BJ** | **TPSS** | **TPSS-D3** | **TPSS-D3BJ** | **MN15L** | **B97-D3** | **B3LYP** | **M11** |
| --- | --- | --- | --- | --- | --- | --- | --- | --- | --- | --- |
| def2-SVP | 6-31G(d) | 16.24 | 16.15 | 17.00 | 16.83 | 16.74 | 15.98 | 16.42 | 17.73 | 15.98 |
|  | 6-31G(d,p) | 16.41 | 15.93 | 17.06 | 16.93 | 16.81 | 15.78 | 16.40 | 17.78 | 16.03 |
|  | 6-31+G(d) | 17.23 | 16.39 | 17.50 | 17.31 | 16.93 | 15.41 | 16.99 | 18.53 | 16.48 |
|  | cc-pVDZ | 16.68 | 16.13 | 17.06 | 16.86 | 16.63 | 16.56 | 16.70 | 17.95 | 16.03 |
|  | jorge-DZP | 16.49 | 16.08 | 17.36 | 17.36 | 17.04 | 16.89 | 16.75 | 17.97 | 16.25 |
|  | Sapporo-DZP-2012 | 16.14 | 15.78 | 16.98 | 16.83 | 16.72 | 16.81 | 16.55 | 17.57 | 16.38 |
|  | def2-SVP | 18.55 | 17.93 | 18.35 | 18.28 | 17.87 | 18.49 | 18.36 | 20.07 | 18.23 |
| LANL2DZ | 6-31G(d) | 14.28 | 13.63 | 15.02 | 14.52 | 14.25 | 14.35 | 13.79 | 15.72 | 13.16 |
|  | 6-31G(d,p) | 14.14 | 13.28 | 14.82 | 14.45 | 14.21 | 14.38 | 13.97 | 15.48 | 13.38 |
|  | 6-31+G(d) | 16.18 | 15.39 | 16.34 | 16.07 | 15.84 | 15.16 | 15.12 | 17.39 | 15.15 |
|  | cc-pVDZ | 14.24 | 13.59 | 14.69 | 14.46 | 14.13 | 14.76 | 14.30 | 15.45 | 13.54 |
|  | jorge-DZP | 14.49 | 14.27 | 15.28 | 15.24 | 15.03 | 15.06 | 14.65 | 16.07 | 13.83 |
|  | Sapporo-DZP-2012 | 13.93 | 13.48 | 14.88 | 14.57 | 14.45 | 14.77 | 14.10 | 15.10 | 13.23 |
|  | def2-SVP | 18.55 | 17.93 | 18.35 | 18.28 | 17.87 | 18.55 | 18.36 | 20.07 | 17.94 |
| Stuttgart-RSC | 6-31G(d) | 14.36 | 14.47 | 15.19 | 15.13 | 14.96 | 14.36 | 14.51 | 15.82 | 13.94 |
|  | 6-31G(d,p) | 14.15 | 14.35 | 15.19 | 15.10 | 14.98 | 14.26 | 14.45 | 15.63 | 14.28 |
|  | 6-31+G(d) | 16.49 | 15.61 | 16.53 | 16.31 | 15.97 | 14.84 | 15.91 | 17.64 | 15.31 |
|  | cc-pVDZ | 14.42 | 14.17 | 15.03 | 14.97 | 14.70 | 14.53 | 14.61 | 15.69 | 13.84 |
|  | jorge-DZP | 15.08 | 14.93 | 16.01 | 15.99 | 15.67 | 15.49 | 15.34 | 16.57 | 14.58 |
|  | Sapporo-DZP-2012 | 13.93 | 14.05 | 15.09 | 15.09 | 14.87 | 14.62 | 14.60 | 15.32 | 13.61 |
|  | def2-SVP | 16.69 | 16.29 | 16.45 | 16.53 | 16.17 | 16.75 | 16.76 | 18.24 | 15.84 |
| Stuttgart-RSC+1f | 6-31G(d) | 14.79 | 14.77 | 15.58 | 15.53 | 15.29 | 14.72 | 14.69 | 16.23 | 14.65 |
|  | 6-31G(d,p) | 14.69 | 14.73 | 15.59 | 15.45 | 15.32 | 14.70 | 14.75 | 16.15 | 15.06 |
|  | 6-31+G(d) | 16.79 | 15.79 | 16.80 | 16.55 | 16.34 | 15.30 | 16.14 | 18.04 | 15.88 |
|  | cc-pVDZ | 14.72 | 14.40 | 15.49 | 15.31 | 15.04 | 14.73 | 14.96 | 16.36 | 14.50 |
|  | jorge-DZP | 15.37 | 15.24 | 16.35 | 13.64 | 16.01 | 15.86 | 15.46 | 16.82 | 14.98 |
|  | Sapporo-DZP-2012 | 14.29 | 14.26 | 15.52 | 12.96 | 15.26 | 14.95 | 14.85 | 16.09 | 14.27 |
|  | def2-SVP | 16.69 | 16.29 | 16.45 | 16.53 | 16.17 | 16.75 | 16.76 | 18.24 | 15.84 |
|  | **Experimental** [2] | **17.76** | | | | | | | | |

**REFERENCES**

[1] Nardin, G., Randaccio, L., Annibale, G., Natile, G., Pitteri, B., *J. Chem. Soc. Dalt. Trans.*, **1980**, 220.

[2] Baddley, W. H., Basolo, F., *Inorg. Chem.*, **1964**, *3*, 1087–1091.
